# Single-cell analyses identify circulating anti-tumor CD8 T cells and markers for their enrichment

**DOI:** 10.1084/jem.20200920

**Published:** 2021-03-02

**Authors:** Kristen E. Pauken, Osmaan Shahid, Kaitlyn A. Lagattuta, Kelly M. Mahuron, Jacob M. Luber, Margaret M. Lowe, Linglin Huang, Conor Delaney, Jaclyn M. Long, Megan E. Fung, Kathleen Newcomer, Katy K. Tsai, Melissa Chow, Samantha Guinn, Juhi R. Kuchroo, Kelly P. Burke, Jason M. Schenkel, Michael D. Rosenblum, Adil I. Daud, Arlene H. Sharpe, Meromit Singer

**Affiliations:** 1 Department of Immunology, Blavatnik Institute, Harvard Medical School, Boston, MA; 2 Evergrande Center for Immunological Diseases, Harvard Medical School and Brigham and Women’s Hospital, Boston, MA; 3 Department of Data Sciences, Dana-Farber Cancer Institute, Boston, MA; 4 Harvard-MIT Medical Scientist Training Program, Harvard Medical School, Boston, MA; 5 Department of Surgery, University of California, San Francisco, San Francisco, CA; 6 Broad Institute of MIT and Harvard, Cambridge, MA; 7 Department of Biomedical Informatics, Harvard Medical School, Boston, MA; 8 Department of Dermatology, University of California, San Francisco, San Francisco, CA; 9 Department of Biostatistics, Harvard H. Chan School of Public Health, Boston, MA; 10 Department of Bioengineering, Northeastern University, Boston, MA; 11 Department of Medicine, University of California, San Francisco, San Francisco, CA; 12 Helen Diller Family Comprehensive Cancer Center, University of California, San Francisco, San Francisco, CA; 13 Department of Medical Oncology, Dana-Farber Cancer Institute and Harvard Medical School, Boston, MA; 14 Koch Institute for Integrative Cancer Research, Massachusetts Institute of Technology, Cambridge, MA; 15 Department of Pathology, Brigham and Women’s Hospital, Boston, MA

## Abstract

The ability to monitor anti-tumor CD8^+^ T cell responses in the blood has tremendous therapeutic potential. Here, we used paired single-cell RNA and TCR sequencing to detect and characterize “tumor-matching” (TM) CD8^+^ T cells in the blood of mice with MC38 tumors or melanoma patients using the TCR as a molecular barcode. TM cells showed increased activation compared with nonmatching T cells in blood and were less exhausted than matching cells in tumors. Importantly, PD-1, which has been used to identify putative circulating anti-tumor CD8^+^ T cells, showed poor sensitivity for identifying TM cells. By leveraging the transcriptome, we identified candidate cell surface markers for TM cells in mice and patients and validated NKG2D, CD39, and CX3CR1 in mice. These data show that the TCR can be used to identify tumor-relevant cells for characterization, reveal unique transcriptional properties of TM cells, and develop marker panels for tracking and analysis of these cells.

## Teaser

Using single-cell RNA sequencing and TCR sequencing, Pauken et al. detect CD8^+^ T cell clones shared between blood and tumor in mice or melanoma patients, characterize matching clones in blood and tumor, and identify potential biomarkers for their isolation in blood.

## Graphical Abstract

**Figure:**
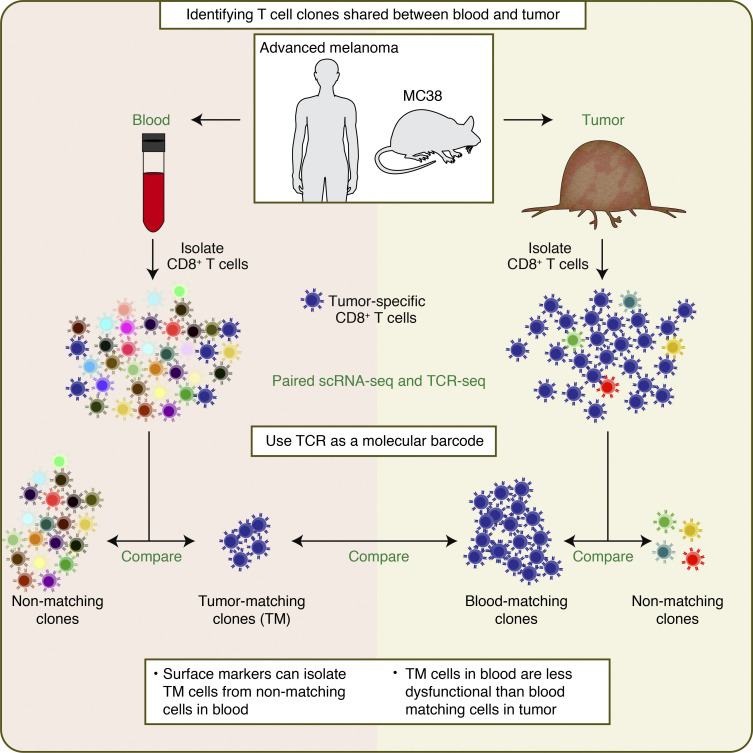

## Introduction

Cancer immunotherapy has revolutionized treatment of many solid and liquid tumors (Chen and Mellman, 2017; Ribas and Wolchok, 2018; Sharma and Allison, 2015; Sharpe and Pauken, 2018; Sun et al., 2018). The systemic immune response is critical for anti-tumor immunity following checkpoint blockade (Fransen et al., 2018; Huang et al., 2019; Huang et al., 2017; Kamphorst et al., 2017; Sharpe and Pauken, 2018; Spitzer et al., 2017; Valpione et al., 2020; Wei et al., 2019; Wu et al., 2020). Recruitment of new CD8^+^ T cells from the circulation into the tumor, termed “clonal replacement,” is associated with better responses to immunotherapy (Cloughesy et al., 2019; Valpione et al., 2020; Wu et al., 2020; Yost et al., 2019). Blood is a major site of CD8^+^ T cell trafficking between secondary lymphoid organs, primary tumors, and metastatic sites (Masopust and Schenkel, 2013), making it an ideal location to interrogate peripheral anti-tumor responses. Studies have profiled T cells in the blood of cancer patients, including during checkpoint blockade (Chalabi et al., 2020; Huang et al., 2019; Huang et al., 2017; Kamphorst et al., 2017; Twitty et al., 2020; Valpione et al., 2020; Wei et al., 2019; Wei et al., 2017; Wu et al., 2020). However, improved methods to identify T cells directed against tumors are needed to focus analyses to the minority of circulating T cells that has prognostic and functional relevance.

Tracking antigen-specific T cells in the blood is difficult because of their small number and limited reagents for detection. Tetramers have been the gold standard for identifying antigen-specific T cells but have limitations, including (a) the antigen must be known; (b) limited available MHC haplotypes for tetramer reagents; and (c) inefficient binding to low-affinity TCRs (Jenkins et al., 2010; Martinez and Evavold, 2015). In humans, surrogate markers like programmed death 1 (PD-1, also known as CD279) and B and T lymphocyte attenuator (BTLA) have been used to enrich the anti-tumor response in blood because of associations with exhaustion in cancer (Gros et al., 2016; Gros et al., 2014; Huang et al., 2019; Huang et al., 2017; Kamphorst et al., 2017; Twitty et al., 2020; Yan et al., 2018). However, PD-1 is not an exhaustion-specific marker. PD-1 is at least transiently expressed on all T cells upon activation, and PD-1^+^ T cells are found in the blood of healthy people (Duraiswamy et al., 2011; Sharpe and Pauken, 2018; Wherry and Kurachi, 2015). Consequently, improving methods to allow routine, unbiased tracking of tumor-specific T cells in blood would bring substantial statistical power and biological precision to analyses of anti-tumor responses.

Here we asked whether single-cell RNA sequencing (scRNAseq) could be used to track tumor-relevant T cell responses in the blood. Using the TCR as a “molecular barcode,” we used paired tumor and blood samples to identify and characterize tumor-matching (TM) blood CD8^+^ T cells that had shared TCR sequences with CD8^+^ T cells in MC38 tumors in mice or melanoma in patients. TM cells generally had an effector/effector memory–like phenotype and appeared less exhausted than clones in tumor. In two longitudinal samples from patients that failed to respond to checkpoint blockade, the TM cells shifted to a stronger dysfunctional signature than before. We identified candidate surface markers that enrich for TM cells and validated three markers using CITE-seq in mice. Importantly, combinations of these marker genes achieved improved performance compared with single markers at identifying TM cells. This work presents an approach to deeply characterize tumor-relevant T cells in blood and identify marker panels to enable focused and statistically powered analyses of such populations.

## Results

### Characterization of CD8^+^ T cells in blood with TCRs that match to CD8^+^ T cells in MC38 tumors

**Figure 1.**
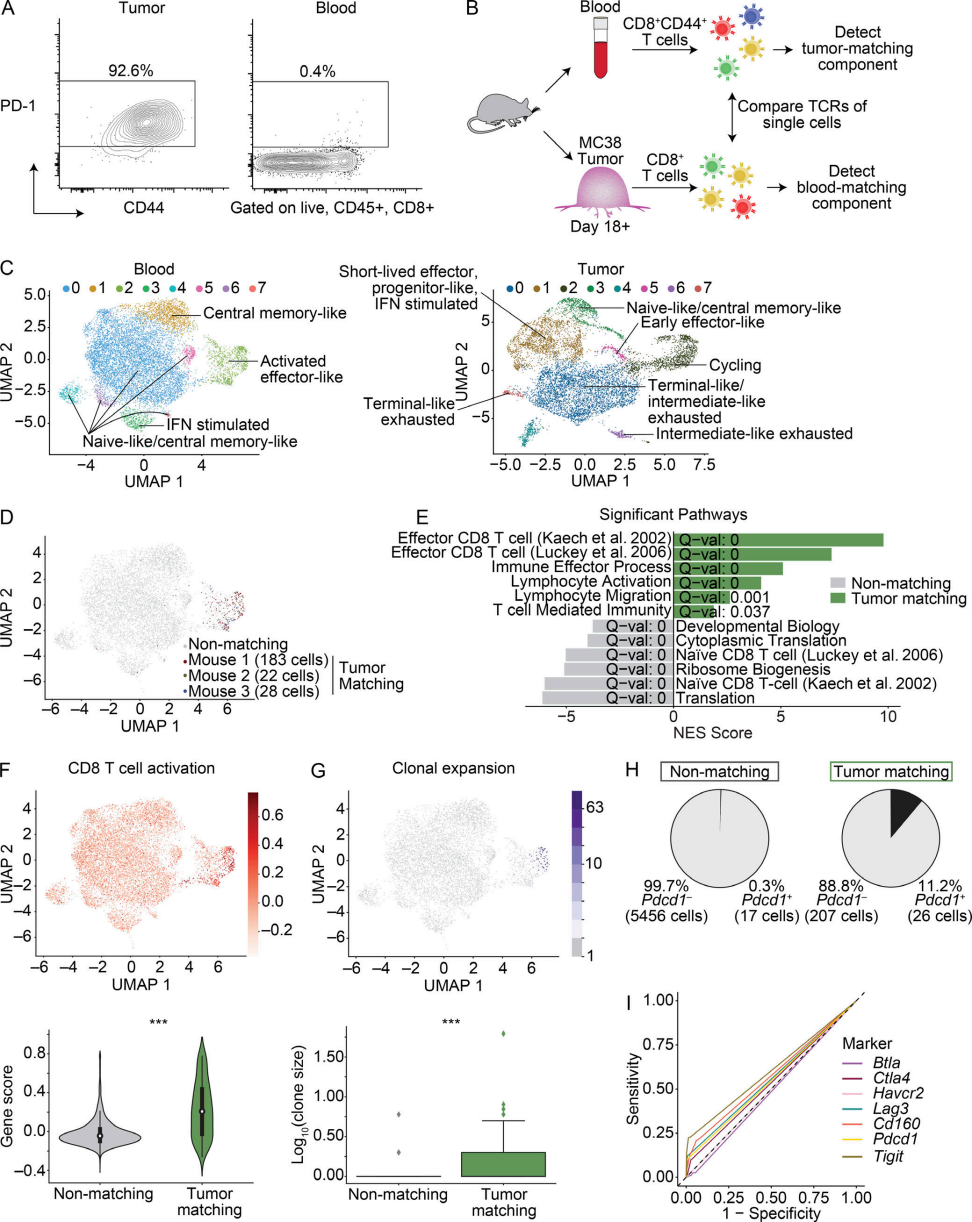
**scRNAseq of CD8^+^ T cells identifies MC38 TM clones in blood based on TCR sequence.** **(A)** FACS plots showing PD-1 and CD44 protein in MC38 tumors and paired blood on day 21. Gated on singlets, live, CD45^+^, CD8α^+^ cells. Frequency of parent expressing PD-1 indicated. Data representative of four experiments, each with *n* = 5–9 mice. **(B)** Experimental design for scRNAseq. **(C)** Clustering and UMAP visualization of paired blood (*n* = 10,289 cells) and MC38 tumors (*n* = 8,450 cells) on day 18+, integrated from three mice (M1–3) from two experiments. Colors denote transcriptional clusters, labeled with functional annotations. **(D)** UMAP showing CD8^+^ T cells in blood that have a TCR matching to CD8^+^ T cells found in tumor (TM cells), colored by each mouse. Gray indicates non-TM cells. **(E)** Selected signatures associated with genes up-regulated in TM cells or non-TM cells in blood. Significance using a gene set enrichment analysis PreRanked analysis. Full list in Table S3. (**(F)** UMAP showing a CD8^+^ activation signature in blood (top). Violin plots of enrichment (bottom). Significance using a Wilcoxon rank sum test, P = 1 × 10^−41^. ***, P < 0.001. **(G)** UMAP showing clonal expansion in the blood (top). Box plot quantifying clonal expansion (bottom). Boxes show the first quartile, median, and third quartile, while the whiskers cover 1.5× the interquartile range. Significance using a Wilcoxon rank sum test, P = 4.6 × 10^−7^. **(H)** Frequency of *Pdcd1*^+^ cells in the blood. **(I)** ROC curve showing the sensitivity and specificity of *Pdcd1*,* Btla*,* Ctla4*,* Havcr2*,* Lag3*,* Cd160*, or *Tigit* to distinguish TM cells from non-TM cells. AUC values: *Pdcd1* = 0.548, *Btla* = 0.486, *Ctla4* = 0.535, *Havcr2* = 0.500, *Lag3* = 0.556, *Cd160* = 0.574, and *Tigit* = 0.603. The dashed line represents the sensitivity and specificity values of random chance. **(C–I)** scRNAseq integrated from three biological replicates (M1–3) from two experiments.

Considering the clinical relevance of tracking anti-tumor CD8^+^ T cells in the blood, we investigated ways to track these cells in tumor-bearing mice. We first assessed PD-1 protein expression on CD8^+^ T cells in mice with subcutaneous colon adenocarcinoma (MC38) tumors. PD-1 levels were uniformly high on CD8^+^ T cells in tumors, but low in the blood (Fig. 1 A), casting doubt on the ability of PD-1 to capture the tumor-relevant CD8^+^ T cell component in blood.

**Figure S1.**
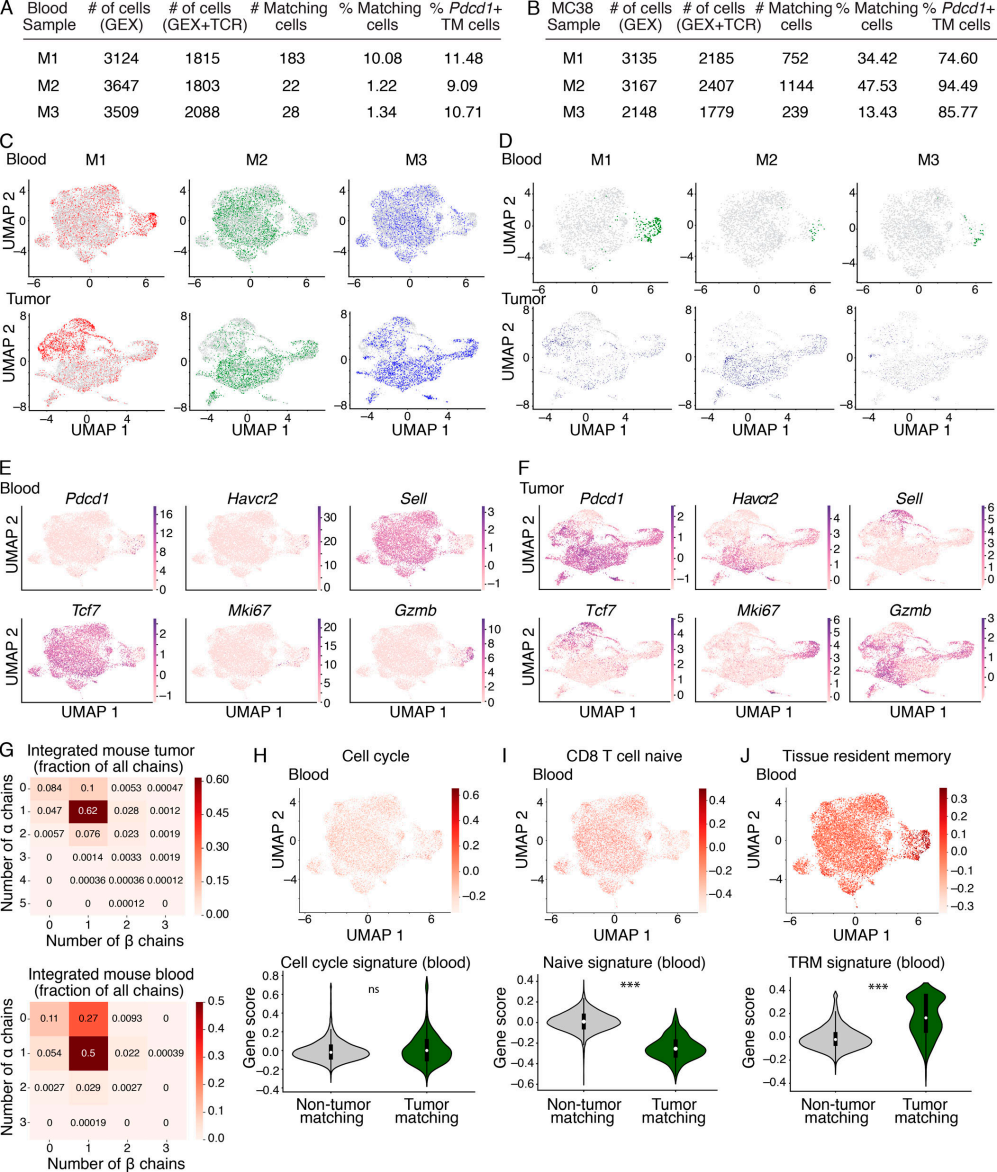
**Transcriptional landscape of CD8^+^ T cells in paired peripheral blood and MC38 tumors in mice.** **(A and B)** Tables indicating details about each mouse in the discovery cohort (M1–3), including the number of cells recovered that had gene expression (GEX) data, GEX and TCR data, number of matching cells, percentage matching cells of the total sorted population, and the frequency of* Pdcd1*^+^ TM cells in peripheral blood (A) and MC38 tumors (B). The samples from M1, M2, and M3 were integrated to generate an integrated blood sample and an integrated MC38 tumor sample as a discovery cohort. These three biological replicates were generated between two independent experiments (M1, experiment 1; M2 and M3, experiment 2). **(C)** UMAP of the integrated blood samples (top) and MC38 tumor samples (bottom) showing the distribution of each mouse in the integrated dataset (datasets combined from M1, M2, and M3). Cells from each mouse are shown in color (M1, red; M2, green; M3, blue), and the cells from the other two mice are shown in gray for each plot. **(D)** UMAP of the integrated blood samples (top) and MC38 tumor samples (bottom) showing the distribution of clones shared between tissues (TM cells in blood, and blood-matching cells in tumor). Only TM cells (green), blood-matching cells (navy blue), and nonmatching cells (gray) from each individual mouse are shown, and the cells from the other two mice in the integrated object are excluded. **(E and F)** UMAPs showing distribution of expression of select transcripts in the integrated blood (E) and MC38 tumor (F) samples. Genes include *Pdcd1* (encoding PD-1), *Havcr2* (encoding Tim-3), *Sell* (encoding CD62L), *Tcf7* (encoding TCF-1), *Mki67* (encoding Ki-67), and *Gzmb* (encoding granzyme B). **(G)** Heatmap showing the fraction of cells in the integrated MC38 tumor (top) and blood (bottom) datasets with the indicated number of TCR α and β chains detected. **(H–J)** Top: UMAP of integrated blood samples showing expression of a cell cycle signature (Kowalczyk et al., 2015; P = 0.24; H), a CD8^+^ naive T cell signature (Kaech et al., 2002; P = 4.6 × 10^−125^; I), and a TRM signature (Beura et al., 2018; P = 5.5 × 10^−60^; J). Violin plots quantifying the expression of each signature in H–J in TM compared with non-TM cells in the blood (bottom). ***, P < 0.001; ns, not significant. Significance determined using Wilcoxon rank sum tests. **(C–J)** scRNAseq integrated from three biological replicates (M1–3) between two independent experiments.

Since the TCR encodes specificity for antigen, we hypothesized that the TCR sequence could be used to assess which clones in blood were relevant to the anti-tumor response. To test this, we performed scRNAseq and TCR sequencing on CD8^+^ T cells isolated from paired blood and MC38 tumors (Figs. 1 B and S1, A–F). The single-cell transcriptomic landscapes of sorted CD44^+^ CD8^+^ T cells in blood (*n* = 10,289 cells; to enrich for rare antigen-experienced cells) and bulk CD8^+^ T cells in tumors (*n* = 8,540 cells) were characterized (Figs. 1 C and S1, E and F). In the blood, most of the cells had a naive-like and/or central memory–like phenotype (Fig. 1 C and Table S1), as expected in specific pathogen–free mice (Beura et al., 2016). Additional phenotypes included recent IFN stimulation and an activated effector-like population (Fig. 1 C and Table S1). In the tumor, more diversity was observed, including progenitor and terminal exhausted subsets (He et al., 2016; Im et al., 2016; Kurtulus et al., 2019; Miller et al., 2019; Sade-Feldman et al., 2018; Siddiqui et al., 2019; van der Leun et al., 2020), as well as an intermediate-like exhausted subset, naive and/or central memory–like cells, effector-like cells, cycling cells, and IFN-stimulated cells (Fig. 1 C and Table S1; Best et al., 2013; Kakaradov et al., 2017; Milner et al., 2017). These data highlight the diversity of CD8^+^ T cell states in MC38 tumors, particularly compared with blood (Fig. 1 C and Table S1).

To assess clonal overlap between blood and tumor, we (a) compared T cells with at least one α and one β chain (Fig. S1 G) and (b) classified cells as the same clone if they exactly matched in their TCR sequences. Using the TCR sequence as a molecular barcode, we observed a population of TM cells in blood that shared TCRs with CD8^+^ T cells in the tumor (Figs. 1 D and S1, A and D). Differentially expressed (DE) gene analysis showed elevated activation markers (e.g., *Ccl5*,* Gzmb*,* Klrg1*,* Klrk1*, and *Cx3cr1*) and decreased naive-like and/or central memory–like markers (e.g., *Ccr7*,* Sell*, and *Tcf7*) in TM cells compared with non-TM cells (Table S2). Pathway enrichment analysis of genes in TM cells showed effector signatures, immune effector processes, and lymphocyte migration, while non-TM cells were enriched for naive CD8^+^ T cell signatures (Fig. 1 E and Table S3). Additionally, using curated signatures from the literature (see Materials and methods), TM cells were enriched for activation and tissue-resident memory (TRM) signatures, while non-TM cells were enriched for a naive signature (Figs. 1 F and S1, I and J; and Table S3). TM cells were also more likely to be clonally expanded (Fig. 1 G), although a signature of cell cycle was low (Fig. S1 H). Importantly, only 11.2% of TM cells expressed the *Pdcd1* transcript (Fig. 1 H). Using receiver operating characteristic (ROC) curves, *Pdcd1* and other inhibitory receptors performed poorly in distinguishing TM cells from non-TM cells, nearing the level of random chance (Fig. 1 I). Collectively, these data are consistent with TM cells actively responding to tumor and support using the TCR to identify TM cells rather than relying on individual markers like PD-1.

### The transcriptional signature of TM CD8^+^ T cells in the blood can be used to identify markers for enrichment via flow cytometry

**Figure 2.**
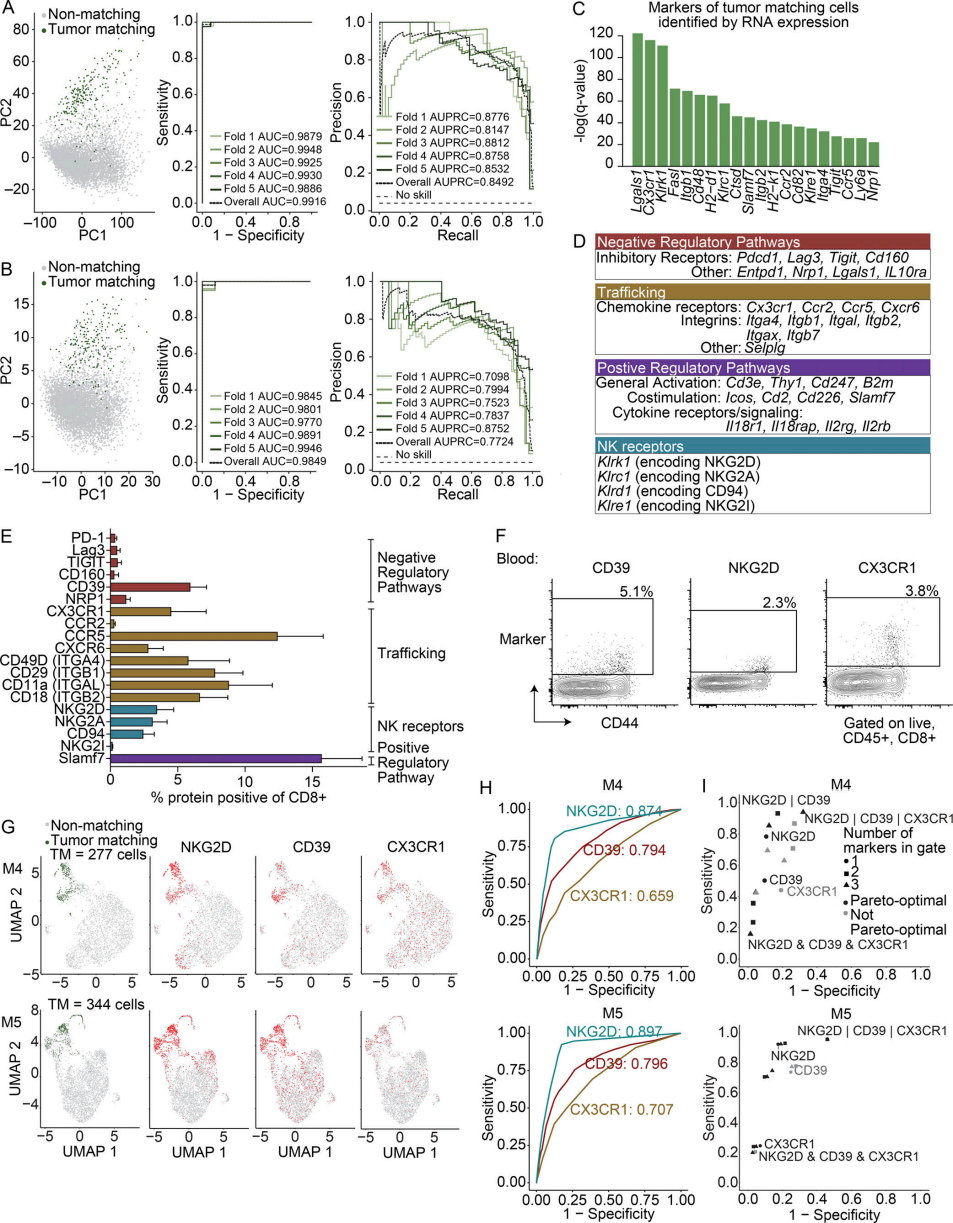
**Cell surface marker panels can enrich TM cells from blood.** **(A and B)** Logistic regression showing classification of cells as TM or non-TM based on (A) all genes and (B) a selected list enriched for surface-marker genes (Chihara et al., 2018). Shown are the first two principal component projections (left), ROC curves (middle), and the recall–precision plots (right) with fivefold cross validation. **(C)** Top 20 surface markers by q value for identifying TM cells in the blood using COMET. Significance using an XL-minimal hypergeometric test with multiple hypothesis test corrections. Full list in Table S4. **(A–C)** scRNAseq integrated from three biological replicates (M1–3) from two experiments. **(D)** Biological functions for positive markers (q value ≤ 0.01) identified using COMET for TM cells. NK, natural killer. **(E)** Frequency protein^+^ of CD8^+^ T cells in the blood of mice with MC38 tumors at day 21 (*n* = 9 mice) by flow cytometry. Gated on singlets, live, CD45^+^, CD8α^+^. Representative of two to four independent experiments depending on the marker, each with *n* = 5–9 mice. Bars show the mean, and error bars represent SD. NK, natural killer. **(F)** FACS plots showing CD39, NKG2D, and CX3CR1 (y axis) as indicated above each plot, and CD44 (x axis) on CD8^+^ T cells in the blood of mice in E. **(G)** UMAP visualization of mice from the validation cohort, two biological replicates, mouse 4 (M4), and mouse 5 (M5). Far left shows cells colored by matching status (green, TM; gray, non-TM). The three UMAPs to the right show cells colored by protein (NKG2D, CD39, and CX3CR1) using CITE seq (red, positive; gray, negative). Significance: CD39, P = 3.87 × 10^−54^ and P = 7.53 × 10^−71^; NKG2D, P = 3.19 × 10^−122^ and P = 1.93 × 10^−175^; CX3CR1, P = 9.22 × 10^−17^ and P = 2.08 × 10^−30^ for M4 and M5, respectively, assessed using Wilcoxon rank sum test. **(H)** ROC curves showing the sensitivity and specificity of each protein at identifying TM cells. **(I)** Sensitivity and specificity for proteins in identifying TM cells as single markers or two- and three-protein combinations, colored black if they are pareto-optimal (no other gate with strictly better sensitivity and specificity) and gray if not pareto-optimal. The “&” indicates an “and” gate, and the “|” indicates an “or” gate. Full list of values in Table S5. **(G–I)** Two biological replicates from the validation cohort (one experiment).

Following our observation that TM cells are transcriptionally distinct, we hypothesized that a machine learning classifier could be trained to predict if a given CD8^+^ T cell from blood is TM or non-TM based on transcriptional data. Indeed, a regularized logistic regression classifier achieved high sensitivity and specificity (Fig. 2 A, cross-validated area under the curve [AUC] = 0.99). We next asked if cell surface genes could distinguish TM from non-TM cells, to assess the potential of identifying cell surface markers for flow cytometry–based sorting for downstream applications. Classifiers using only a list of cell surface genes (Chihara et al., 2018) also achieved high sensitivity and specificity (Fig. 2 B, cross-validated AUC = 0.985).

**Figure S2.**
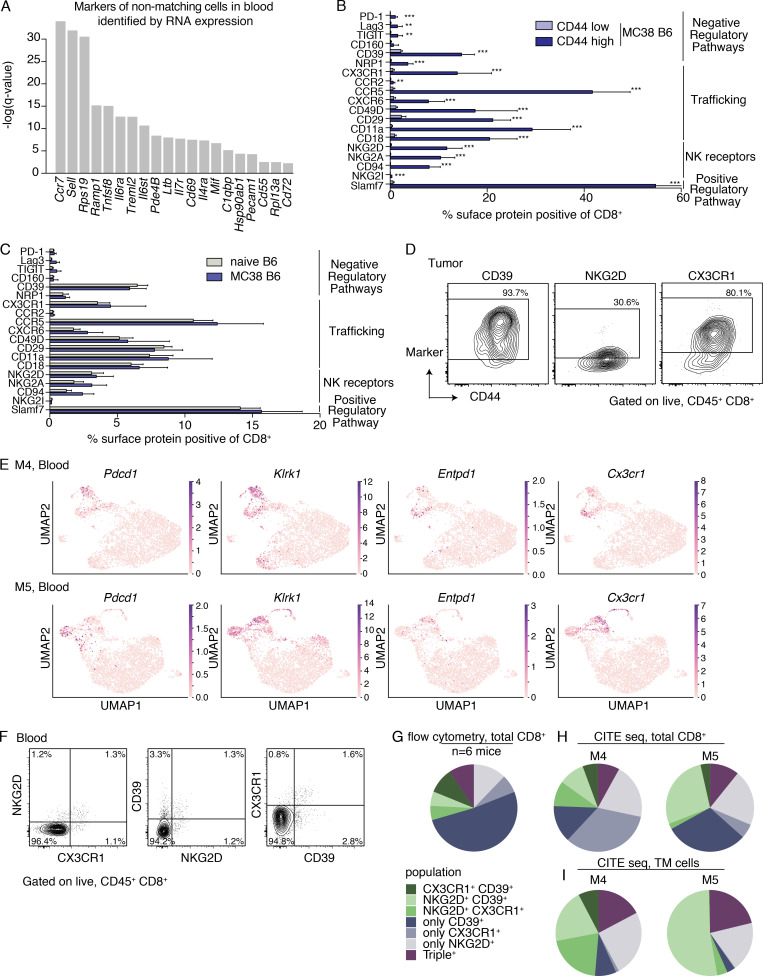
**Identification and validation of markers to identify TM CD8^+^ T cells in blood** **(A)** Top surface markers for identifying non-TM cells from TM cells in the blood based on COMET (Delaney et al., 2019) analysis. Significance determined using an XL-minimal hypergeometric test with multiple hypothesis test corrections. scRNAseq integrated from three biological replicates (M1–3) between two independent experiments. **(B)** Quantification of the frequency of bulk CD8^+^ T cells in the peripheral blood of mice with MC38 tumors on day 21 after implantation (*n* = 9 mice) that express the indicated proteins using FACS. Cells are gated on singlets, live/dead^−^, CD45^+^, and CD8α^+^ and are further gated based on CD44 expression to compare CD44^low^ and CD44^high^ cells. **, P < 0.01; ***, P < 0.001. **(C)** Comparison of bulk CD8^+^ T cells (gated on singlets, live/dead^−^, CD45^+^, CD8α^+^) from the peripheral blood of mice with MC38 tumors on day 21 after implantation (*n* = 9 mice) to naive B6 mice (*n* = 4 mice). **(B and C)** Data are representative of two to four independent experiments depending on the marker, with *n* = 3–4 naive mice and *n* = 5–9 mice with MC38 tumors (days 19–22). Bars show the mean, and error bars represent SD. Significance determined using multiple *t* tests using the Holm-Sidak method, with α = 0.05. Each row was analyzed individually, without assuming a consistent SD. Reported are the adjusted P values considering multiple tests. Significant comparisons in B are indicated with asterisks and include PD-1, P = 2.6957 × 10^−5^; Lag-3, P = 0.0012; TIGIT, P = 0.0012; CD39, P = 3.7639 × 10^−9^; NRP1, P = 5.1172 × 10^−7^; CX3CR1, P = 0.0002; CCR2, P = 0.0012; CCR5, P = 6.4414 × 10^−10^; CXCR6, P = 6.1903 × 10^−5^; CD49, P = 0.0002; CD29, P = 1.6745 × 10^−9^; CD11a, P = 1.1636 × 10^−7^; CD18, P = 1.9906 × 10^−7^; NKG2D, P = 1.1863 × 10^−7^; NKG2A, P = 1.8567 × 10^−7^; CD94, P = 1.8567 × 10^−7^; NKG2I, P = 2.6625 × 10^−6^; Slamf7, P = 5.06 × 10^−13^. In B, CD160 expression between CD44^high^ and CD44^low^ was not significant. In C, there were no significant differences between naive B6 and B6 mice with MC38 tumors. **(D)** Representative FACS contour plots showing NKG2D, CD39, and CX3CR1 expression (y axis) as indicated above each plot and CD44 (x axis) on CD8^+^ T cells in the MC38 tumor of mice in Fig. 2 F. Representative of three independent experiments, each with *n* = 5–9 mice. **(E)** UMAPs showing distribution of expression of select transcripts in the blood of M4 (top) and M5 (bottom), two biological replicates from the validation cohort (one experiment). Genes include *Pdcd1* (encoding PD-1), *Klrk1* (encoding NKG2D), *Entpd1* (encoding CD39), and *Cx3cr1* (encoding CX3CR1). **(F)** Representative FACS contour plots showing all possible pairwise combinations of NKG2D, CD39, and CX3CR1 expression (as indicated in each plot) on CD8^+^ T cells in the blood of mice on day 21 after implantation of MC38 tumor cells. Plots are gated on singlets, live/dead^−^, CD45^+^, CD8α^+^. Numbers on plots indicate the percentage of cells within each quadrant of the total parent population. **(G)** Quantification of the flow cytometry plots in F showing the frequency of cells expressing one, two, or three of the indicated proteins (NKG2D, CD39, and CX3CR1) determined using Boolean gating, of the population of cells expressing at least one of the markers. Shown are the average frequencies of all possible combination gates from six mice. In G, 70.5% expressed only one of the markers but not the others, 20.1% expressed only two of the markers, and 9.4% expressed all three of the markers. Data in F and G are representative of three independent experiments with five to nine mice per experiment. **(H and I)** Quantification of the frequencies of cells expressing one, two, or three of the indicated proteins (NKG2D, CD39, and CX3CR1) of the population of cells expressing at least one of the markers in the blood of M4 and M5 using the CITE seq data (two biological replicates from the validation cohort; one experiment). The frequencies of all possible combination gates on the total population of cells from the CITE seq experiment (not subsetting based on TM status; H) and only the TM population (I). **(H)** 61.9% of cells expressed only one marker, 28.7% expressed only two markers, and 9.4% expressed all three markers (values averaged between M4 and M5). **(I)** 28.1% of cells expressed only one marker, 52.7% expressed only two markers, and 19.2%, expressed all three markers (values averaged between M4 and M5). The pie charts in G and H share the legend to the left of I.

To test whether single-gene surface markers could identify the TM component, we applied COMET, a computational tool we developed to predict markers from scRNAseq data (Delaney et al., 2019). COMET identified 82 candidate positive markers for the TM component (with q value ≤0.01) classified into four general biological categories: negative regulatory pathways, positive regulatory pathways, trafficking molecules, and natural killer receptors (Fig. 2, C and D; and Table S4). COMET also identified 21 candidate positive markers associated with non-TM cells (Fig. S2 A), many consistent with their naive and/or central memory–like phenotype (e.g., *Ccr7*,* Sell*, and* Il7r*; Figs. 1 E and S1 I).

Several candidate markers were also detected at the protein level (Fig. 2 E) and were enriched on CD44^+^ cells (Fig. S2 B). Some markers trended toward a higher frequency in the blood of mice bearing MC38 tumors than naive mice, but many including PD-1 were not different (Fig. S2 C). To test if surface proteins could enrich for TM cells, we evaluated three of the COMET-predicted candidates (*Entpd1* encoding CD39, *Cx3cr1* encoding CX3CR1, and *Klrk1* encoding NKG2D; Fig. 2 D and Table S4). A small number of CD8^+^ T cells expressed these proteins in the blood of mice with MC38 tumors (Fig. 2, E and F), albeit less than observed in the tumor (Fig. S2 D). We next performed a scRNAseq experiment measuring gene expression, TCR, and protein expression for CD39, CX3CR1, and NKG2D using CITE-seq (Stoeckius et al., 2017) in two mice (Figs. 2 G and S2 E) to determine if these proteins could enrich for TM cells identified using the TCR. As single markers, each protein successfully enriched for TM cells (Fig. 2, G and H). We next asked if combinations were useful for identifying TM cells. Most TM cells expressed two or three of the markers (Fig. S2, F–I). Moreover, using combinations improved on either or both the sensitivity and specificity over single markers (Fig. 2 I and Table S5). Consequently, while the TCR likely remains the most sensitive and specific metric for determining whether T cells have shared reactivity, cell surface markers can be identified and used to distinguish TM cells from non-TM cells.

### TM CD8^+^ T cells in blood are less dysfunctional than matching clones found in tumor

**Figure S3.**
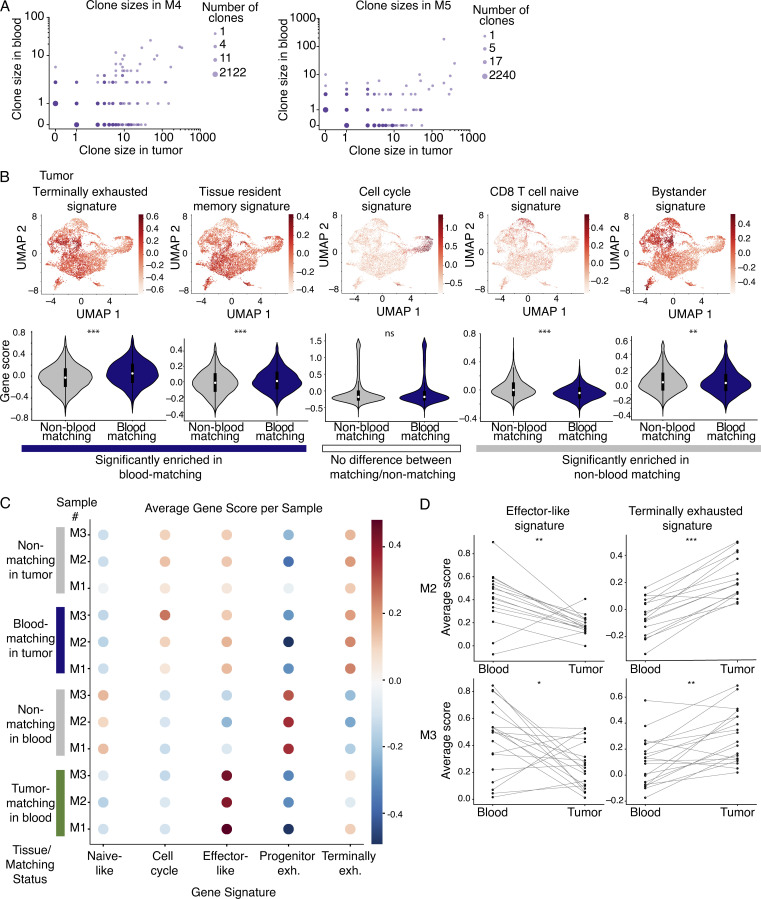
**TM CD8^+^ T cells in the blood show stronger enrichment for effector signatures and weaker enrichment for exhaustion signatures than the corresponding clones in the tumor.** **(A)** Expansion rates of clones in blood and MC38 tumor (log scale), for M4 (left) and M5 (right). Shown are two biological replicates from the validation cohort (one experiment). Data from M1 from an independent experiment are shown in Fig. 3 D. **(B)** Top: UMAP visualization of signatures related to CD8^+^ T cell transcriptional states in the mouse integrated MC38 tumor samples. From left to right are signatures of terminal exhaustion from Miller et al. (2019); TRM cells from Beura et al. (2018); cell cycle from Kowalczyk et al. (2015); naive cells from Kaech et al. (2002); and bystander cells with TCRs that are not specific to the tumor from Mognol et al. (2017). Bottom: Violin plots quantifying the expression of each signature in blood-matching compared with non–blood matching clones. Significance determined using a Wilcoxon rank sum test. Colored bars beneath the violin plots indicate whether the mean is statistically greater in blood-matching cells (terminal exhaustion, P = 1 × 10^−41^; TRM P = 6.9 × 10^−13^), not statistically significant (cell cycle, P = 0.97), or statistically greater in non–blood-matching cells (naive, P = 2.2 × 10^−65^; bystander, P = 0.0016). scRNAseq integrated from three biological replicates (M1–3) between two independent experiments. **(C)** Shown are average gene scores per sample for mouse blood and tumor, separated by matching status. M1–3 indicate each mouse sample number (three mice between two independent experiments). For a given signature, a gene score was calculated for each cell. Shown are naive-like (Kaech et al., 2002), cell cycle (Kowalczyk et al., 2015), and the effector-like, progenitor, and terminally exhausted signatures from Miller et al. (2019). **(D)** Clone-by-clone analysis examining the mean expression of an effector-like gene signature or a terminal exhaustion gene signature from Miller et al. (2019). Each dot shows the average gene signature of the cells in a given clone, and lines connect the same clone between blood and tumor samples. Shown are clones detected in M2 (top) and M3 (bottom) from one experiment. Data from M1 from an independent experiment are shown in Fig. 3 E. Significance determined using a Wilcoxon signed-rank test. For M2, P = 0.0084 for the effector-like signature and P = 5.3 × 10^−4^ for the terminally exhausted signature. For M3, P = 0.024 for the effector-like signature, and P = 1.7 × 10^−3^ for the terminally exhausted signature. *, P< 0.05; **, P < 0.01; ***, P < 0.001; ns, not significant.

**Figure 3.**
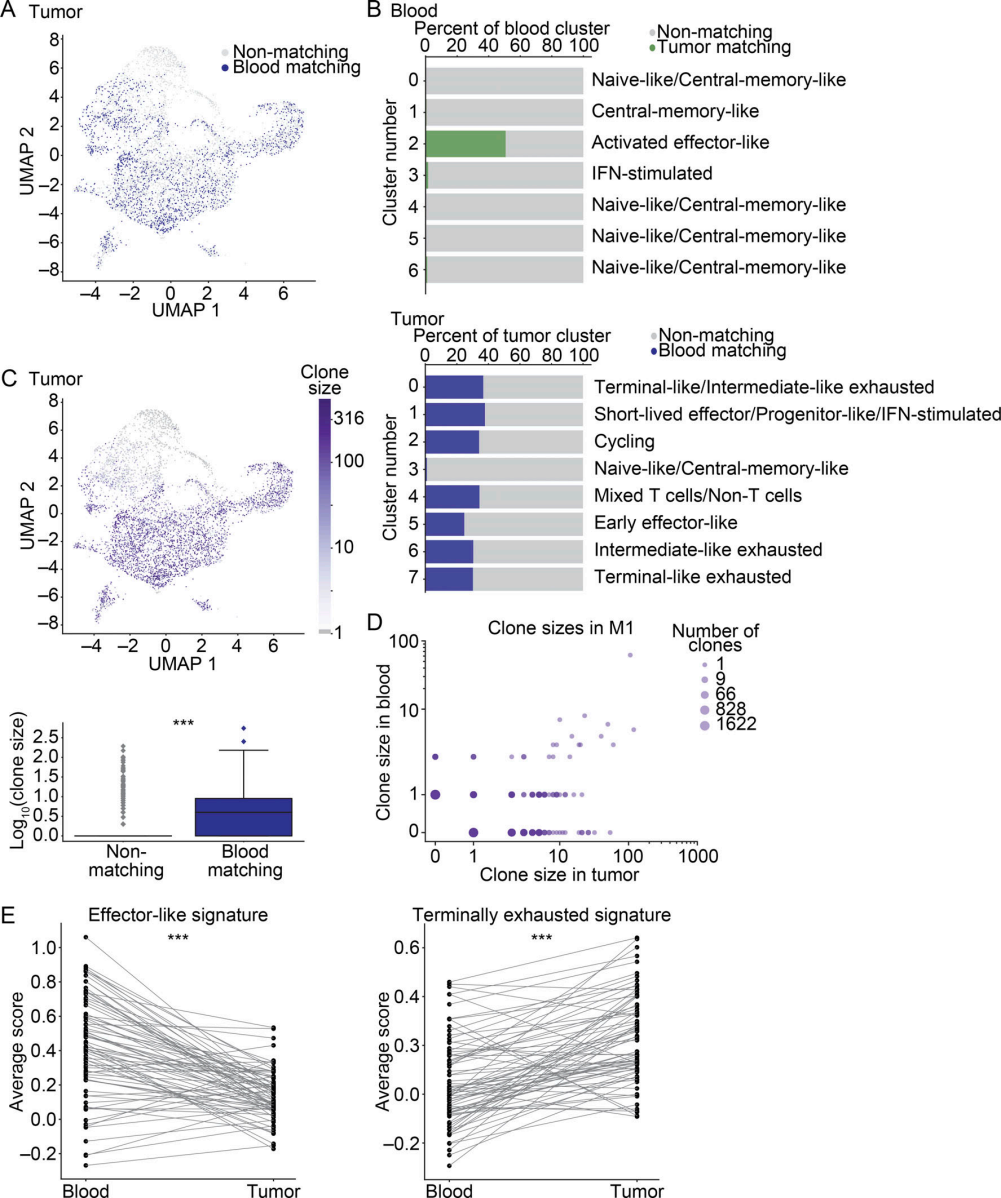
**TM CD8^+^ T cells in blood are less dysfunctional than the corresponding clones in tumor.** **(A)** CD8^+^ T cells from the integrated MC38 tumor samples colored by matching status. Navy blue, blood-matching cells; gray, nonmatching cells. **(B)** The distribution of cells in blood (top) and MC38 tumors (bottom). Shown is the percentage of each cluster that is matching versus nonmatching. Shown are clusters with >50 cells. **(C)** UMAP visualization showing clone size across in tumor (top). Box plot quantifying clonal expansion in the tumor (bottom). Significance using the Wilcoxon rank sum test, P = 4.9 × 10^−26^. **(A–C)** scRNAseq integrated from three biological replicates (M1–3) from two experiments. **(D)** Expansion rates of clones in blood and MC38 tumor (log-scale, for M1). Shown is M1 (one experiment), analysis of M4 and M5 from an independent experiment shown in Fig. S3 A. **(E)** Enrichment scores for a terminal exhaustion signature (P = 1.9 × 10^−9^) and an effector-like signature (P = 1.3 × 10^−9^) in tumor and blood. Significance using a Wilcoxon signed-rank test. Each dot shows the average gene signature of the cells in a given clone, and lines connect the same clone between tissues. Shown are clones detected in M1 (one experiment). M2 and M3 from an independent experiment shown in Fig. S3 D. ***, P < 0.001.

We next examined the transcriptional heterogeneity of CD8^+^ T cells in the tumor whose TCRs were also detected in blood, referred to as “blood-matching” cells. Blood-matching cells were present in every transcriptional cluster in the tumor (Fig. 3, A and B; and Fig. S1 D), with the majority present in nonnaive/noncentral memory–like clusters (Fig. 3 B). Blood-matching cells were more clonally expanded than nonmatching cells (Fig. 3 C, P = 4.9 × 10^−26^). In M1, clone size in blood correlated with clone size in tumor (Fig. 3 D). While clone sizes were too low in M2 and M3 to observe a significant correlation in expansion between blood and tumor, we did observe this correlation in the two mice in our validation cohort (M4 and M5) where the number of TM cells recovered was higher (Fig. S3 A).

To further characterize blood-matching T cells in the tumor, we examined signatures related to CD8^+^ T cell functions. Compared with nonmatching cells, blood-matching cells expressed higher levels of a terminal exhaustion signature and a TRM signature, associated with TRM cells, which can play a role in protective anti-tumor immunity (Menares et al., 2019; Park et al., 2019; Fig. S3 B). Blood-matching cells expressed lower levels of a naive T cell signature, and no difference was observed in a cell cycle signature (Fig. S3 B). Lastly, pathogen-specific CD8^+^ T cells can infiltrate tumors in both mice and humans (Mognol et al., 2017; Rosato et al., 2019; Simoni et al., 2018). This bystander transcriptional signature (Mognol et al., 2017) was observed in MC38 tumors (Fig. S3 B) but was expressed at lower levels in blood-matching cells compared with nonmatching cells. These findings suggest that the TM component in blood corresponds to matching clones in the tumor that are likely responding to tumor antigens and relevant for tumor killing.

Next, we compared the transcriptional profiles of TM cells in blood to matching clones in the tumor. The blood-matching population within tumor was more diverse than the TM population in blood (Fig. 3 B), suggesting that CD8^+^ T cells can diversify and take on a number of states upon entering tumors. On both a population level (Fig. S3 C) and a clone-by-clone basis (Figs. 3 E and S3 D), TM cells were significantly more enriched for an effector-like signature than blood-matching cells in tumor, and blood-matching cells in the tumor were more enriched for the terminal exhaustion signature than TM cells in blood (Figs. 3 E and S3 D). Additionally, DE gene analysis on clonally matched populations between blood and tumor showed many effector-like genes up-regulated in TM clones in the blood (e.g., *Ccl5*,* Cx3cr1*,* Itga4*,* Runx1*, and *Klrg1*) and exhausted-like genes up-regulated in the blood-matching clones in tumor (e.g., *Pdcd1*,* Lag3*,* Ctla4*,* Havcr2*, and* Tigit*; Table S6). Clones in tumors also showed elevated levels of many of the granzymes (*Gzmb*,* Gzmc*,* Gzmf*, and* Gzmg*; Table S6), consistent with work showing some overlap between effector-associated genes and exhausted T cell populations, particularly terminally exhausted T cells (Beltra et al., 2020; Singer et al., 2016). These data suggest that TM cells in the blood are less dysfunctional than their counterparts in tumor, and that after migration into the tumor, these TM cells acquire a dysfunctional state.

### Activated TM CD8^+^ T cells can be detected in the blood of melanoma patients

**Figure S4.**
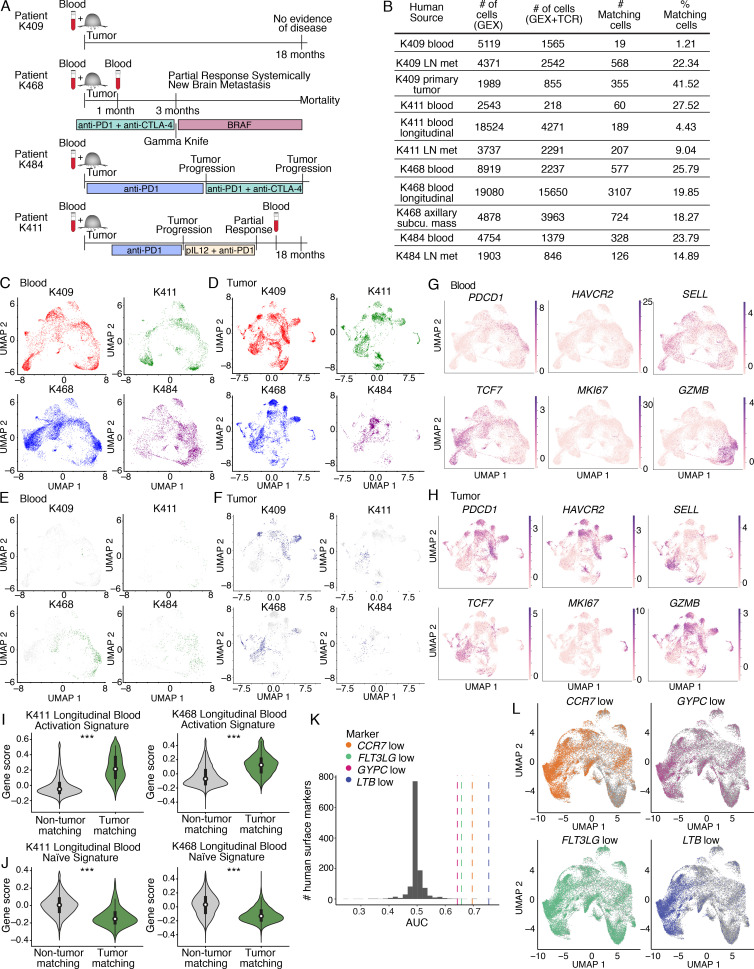
**Transcriptional landscape of CD8^+^ T cells in paired patient peripheral blood and melanoma samples.** **(A)** Schematic of clinical parameters for patient samples. Patients were checkpoint-treatment naive at the time of initial paired blood/tumor sampling. Subsequent course of treatment indicated. Timing of longitudinal blood sample collection for follow-up analysis in patients K468 and K411 indicated. The longitudinal sample for K468 was taken 1 mo after the initial blood sample, and during that time the patient received anti–PD-1 and anti–CTLA-4 combination therapy. The longitudinal sample for K411 was taken ~16 mo after the initial sample, after the patient had received anti–PD-1 as a single agent followed by combination therapy with anti–PD-1 and tavokinogene telseplasmid (TAVO; Algazi et al., 2020 for TAVO monotherapy, and clinicaltrials.gov reference NCT03132675 for combination). **(B)** Table indicating details regarding each patient in the cohort, including the site of tissue resection, number of cells recovered that had gene expression (GEX) data, GEX and TCR data, number of matching cells, percentage matching cells of the total sorted population, and the frequency of *PDCD1*^+^ TM cells. Each patient and time point was processed as an independent experiment for a total of six experiments (four treatment-naive blood/tumor pairs and two longitudinal blood follow-up samples). **(C–F)** UMAP visualization of the integrated initial paired blood samples (C and E) and melanoma samples (D and F) showing the distribution of each patient in the integrated object. Cells are colored by patient, and the remaining cells in the integrated object are excluded from visualization. C and D indicate all cells from a given patient; E and F show matching cells colored in green (TM cells in blood) or navy blue (blood-matching cells in tumor) and nonmatching cells in gray from each patient. **(G and H)** UMAP visualizations showing the distribution of expression of select transcripts in the integrated blood (G) and melanoma (H) samples. Genes include *PDCD1* (encoding PD-1), *HAVCR2* (encoding Tim-3), *SELL* (encoding CD62L), *TCF7* (encoding TCF-1), *MKI67* (encoding Ki-67), and *GZMB* (encoding granzyme B). The data in C–H are integrated from the four initial blood/tumor samples, totaling four independent experiments. **(I and J)** Violin plots showing expression of activation (I) or naive (J) CD8^+^ T cell signatures in TM and non-TM cells in the longitudinal blood samples from K411 and K468. Signatures derived from Akondy et al. (2017). Significance determined used a Wilcoxon rank sum test. For the activation signature in I, P = 1.2 × 10^−76^ for K411 and P < 0.001 for K468. For the naive signature in J, P = 6.5 × 10^−52^ for K411 and P < 0.001 for K468. Each longitudinal patient sample was collected and run separately, totaling two independent experiments. ***, P < 0.001. **(K)** Histogram showing the distribution of AUC values averaged across the six patient samples for each of the human surface markers (Chihara et al., 2018) as positive or negative indicators of TM status. Colored lines represent the AUC for *CCR7*, *FLT3LG*, *GYPC*, and *LTB* averaged across the six patient samples as negative indicators of TM status. **(L)** UMAP visualizations of the top singleton marker gates in human in CD8^+^ cells from all six patient blood samples integrated as described in Materials and methods. In each plot, cells are colored if they pass the particular negation gate; that is, if they are selected as TM because of their low expression of the marker (labeled marker^low^). For *CCR7^low^*, sensitivity = 0.827, specificity = 0.619; for *FLT3LG^low^*, sensitivity = 0.780, specificity = 0.447; for *GYPC^low^*, sensitivity = 0.339, specificity = 0.819; for *LTB ^low^*, sensitivity = 0.725, specificity = 0.716. K and L show the data integrated for all six blood samples (four initial treatment-naive samples and two longitudinal follow-up samples), totaling six independent experiments. For patient samples, “tumor” in the figure refers to resections from the primary tumor and/or metastases as indicated in Fig. S4 B.

**Figure 4.**
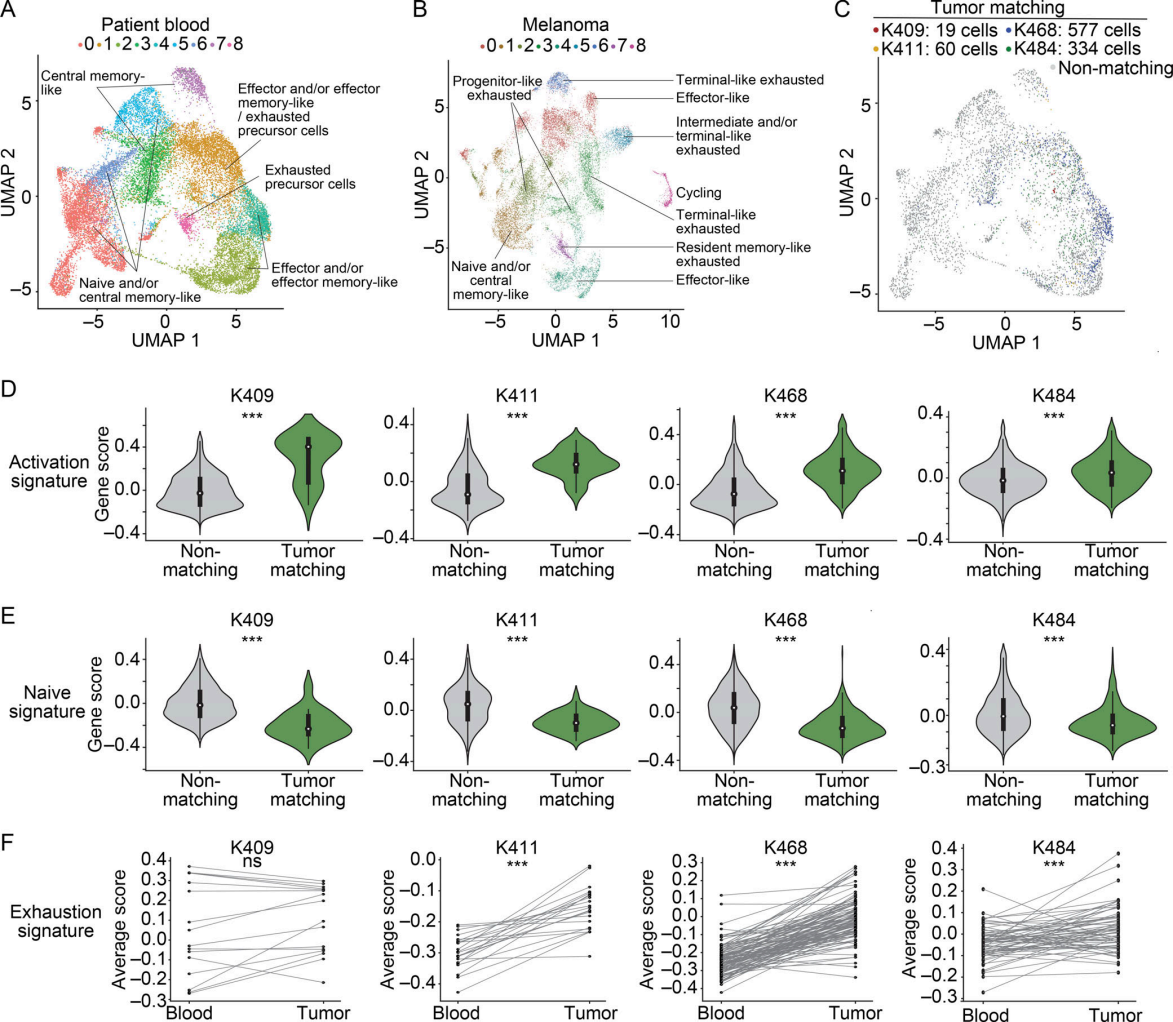
**TM CD8^+^ T cell clones can be detected in the blood of metastatic melanoma patients and show fewer signs of dysfunction than matching clones in tumor.** **(A and B)** Clustering and UMAP visualization of paired blood (*n* = 21,833 cells) and tumor (*n* = 16,878 cells) samples from immunotherapy treatment naive patients, filtered to show CD8^+^ T cells. Data are integrated from four patients (four experiments, patient clinical parameters in Fig. S4 A and Table S7). Colors indicate transcriptional clusters. Functional annotations of each cluster are indicated. **(C)** CD8^+^ T cells in blood colored by matching status in each patient (color, TM; gray, non-TM). **(D and E) **Enrichment of activation (D) or naive (E) CD8^+^ T cell signatures. Significance using a Wilcoxon rank sum test. For D, P values are K409, P = 7 × 10^−8^; K411, P = 3.3 × 10^−15^; K468, P = 3.2 × 10^−101^; K484, P = 3.4 × 10^−13^. For E, P values are K409, P = 1.4 × 10^−7^; K411, P = 2.3 × 10^−10^; K468, P = 1.6 × 10^−91^; K484, P = 1.4 × 10^−12^. **(F)** Mean value of an exhaustion signature in blood and in tumor. Significance using a Wilcoxon signed-rank test; P values are K409, P = 0.2; K411, P = 4 × 10^−5^; K468, P = 8.9 × 10^−19^; K484, P = 6.7 × 10^−5^. Each dot shows a clone, and lines connect the same clone between tissues. For patients, “tumor” refers to resections from the primary tumor and/or metastases as indicated in Fig. S4 B. **(D–F)** Four independent experiments. ***, P < 0.001; ns, not significant.

We next performed scRNAseq and TCR sequencing on four checkpoint treatment-naive advanced melanoma patients (Fig. S4, A–H; and Table S7). Here, “tumor” refers to tissue resections obtained from the primary tumor site and/or metastases (Fig. S4 B and Table S7). CD8^+^ T cells had transcriptional signatures in blood consistent with naive-like, central memory–like, effector-like, and effector memory–like cells and signatures in tumor consistent with diverse exhausted subpopulations, effector-like, resident memory–like, naive-like and/or central memory–like, and cycling populations (Fig. 4, A and B; and Table S8), consistent with previous reports (Guo et al., 2018; Sade-Feldman et al., 2018; Siddiqui et al., 2019; Tirosh et al., 2016; van der Leun et al., 2020; Yost et al., 2019).

Next, we detected TM cells in the blood using the TCR sequence as a molecular barcode (Figs. 4 C and S4 B). Despite heterogeneity across patients (Fig. S4, E and F), the majority of TM cells in each patient were present in nonnaive clusters (e.g., all clusters except clusters 0, 5, and 6; Fig. 4, A and C). The percentages of TM cells in these nonnaive clusters were 89.5% (K409), 100% (K411), 99.7% (K468), and 96.6% (K484). TM cells mostly belonged to clusters associated with an effector and/or effector memory–like phenotype (clusters 1, 2, and 4; Fig. 4, A and C). Consistent with this, TM cells in the blood expressed significantly higher levels of an activation signature compared with non-TM cells (Fig. 4 D), and non-TM cells expressed significantly higher levels of a naive signature (Fig. 4 E). To interrogate how the level of exhaustion compared between clones in blood and clones in tumor, we evaluated an exhaustion signature on a clone-by-clone basis between these two tissues. In patient K409, there was no significant difference in the exhaustion score between clones in blood and tumor (Fig. 4 F, K409, P = 0.2). However, in the other three patients analyzed, the exhaustion signature was significantly elevated on matching clones in tumor relative to blood (Fig. 4 F, K411, P = 4 × 10^−5^; K468, P = 8.9 × 10^−19^; K484, P = 6.7 × 10^−5^). These data are consistent with our results in mice, supporting the idea that TM cells in the blood may be less dysfunctional than their corresponding counterparts in tumor.

### TM CD8^+^ T cells can be tracked longitudinally in patient blood and show a temporal increase in exhaustion despite anti–PD-1 treatment

**Figure 5.**
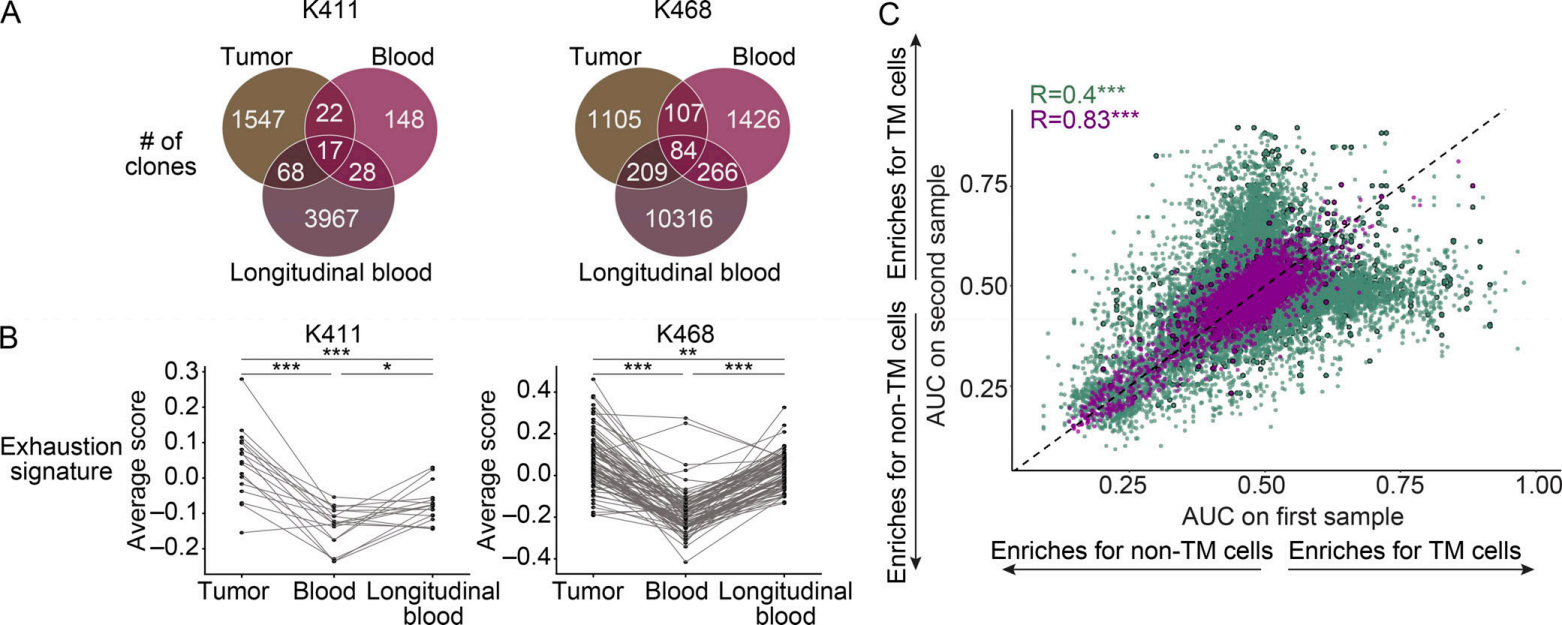
**Matching clones can be detected in longitudinal blood samples from melanoma patients.** **(A)** Number of clones detected and overlapping between samples in the initial blood, longitudinal blood, and tumor samples of K411 and K468 (two experiments). **(B)** Mean value of an exhaustion gene signature from tumor, initial paired blood, and longitudinal blood. Each dot shows a clone, and lines connect the same clone between samples. Shown are only clones that were detectable in all samples. Significance using a Wilcoxon signed-rank test. For patient K411, blood versus longitudinal blood, P = 0.025; blood versus tumor, P = 3.5 × 10^−4^; longitudinal blood versus tumor, P = 0.001. For patient K468, blood versus longitudinal blood, P = 1.2 × 10^−14^; blood versus tumor, P = 6 × 10^−15^; longitudinal blood versus tumor, P = 0.0015. *, P < 0.05; **, P < 0.01; ***, P < 0.001. **(C)** Scatter plot showing each gene’s AUC for selecting TM cells from blood. Purple is a comparison between longitudinal samples from the same patient (*R* = 0.4, P < 2.2 × 10^−16^). Green is a comparison between different patients (*R* = 0.83, P < 2.2 × 10^−16^). Points outlined in black are surface-expressed genes. ***, P < 0.001. Significance using the Spearman correlation test. For patients, “tumor” refers to resections from the primary tumor and/or metastases as indicated in Fig. S4 B.

Follow-up blood samples were obtained from two patients that failed to respond to checkpoint blockade, K411 and K468 (Fig. S4 A). We detected overlapping TCRs between the two blood samples and the tumor sample in each patient, despite one of the samples being collected almost a year and half after the initial sample (Figs. 5 A and S4 A). TM cells detected in the longitudinal samples showed increased activation compared with non-TM cells (Fig. S4, I and J), similar to the trend in the initial sample (Fig. 4, D and E). Notably, the exhaustion signature was higher in the longitudinal samples than the initial blood samples, but lower than the tumor (Fig. 5 B). These data suggest that TM cells in the blood can become more exhausted over time despite anti–PD-1 treatment, but ultimately the highest levels of exhaustion were in the tumor.

We next quantified the extent to which the transcripts enriched in the TM component relative to the non-TM component correlated across patient samples. The extent of similarity across samples was greater for within-patient comparisons than between-patient comparisons (Fig. 5 C and Table S9). Despite the acquired differences in the T cell exhaustion signature of clones following therapeutic intervention (Fig. 5 B), the general transcriptional landscape of the TM component relative to the non-TM component remained highly consistent within the two patients assessed in this study (Fig. 5 C; *R* = 0.83, P < 2.2 × 10^−16^).

Analysis of between-patient variability revealed a significant correlation (Fig. 5 C; *R* = 0.4, P < 2.2 × 10^−16^) in the extent to which individual gene transcripts were specific to the TM component or the non-TM component. This consistency suggested there may be useful transcripts for isolating the TM component from blood. We therefore restricted our correlation analysis to cell surface markers (Chihara et al., 2018), since their transcripts would have practical uses (e.g., sorting for sequencing, functional assays, or adoptive cell transfer therapy), and correlations in the TM component remained (*R* = 0.31, P < 2.2 × 10^−16^). This result suggests that surface-expressed biomarkers could be defined for the TM population that are robust to varying tumor burdens and therapeutic conditions.

### Cell surface marker combinations can be used to detect the TM component from patient blood

**Figure 6.**
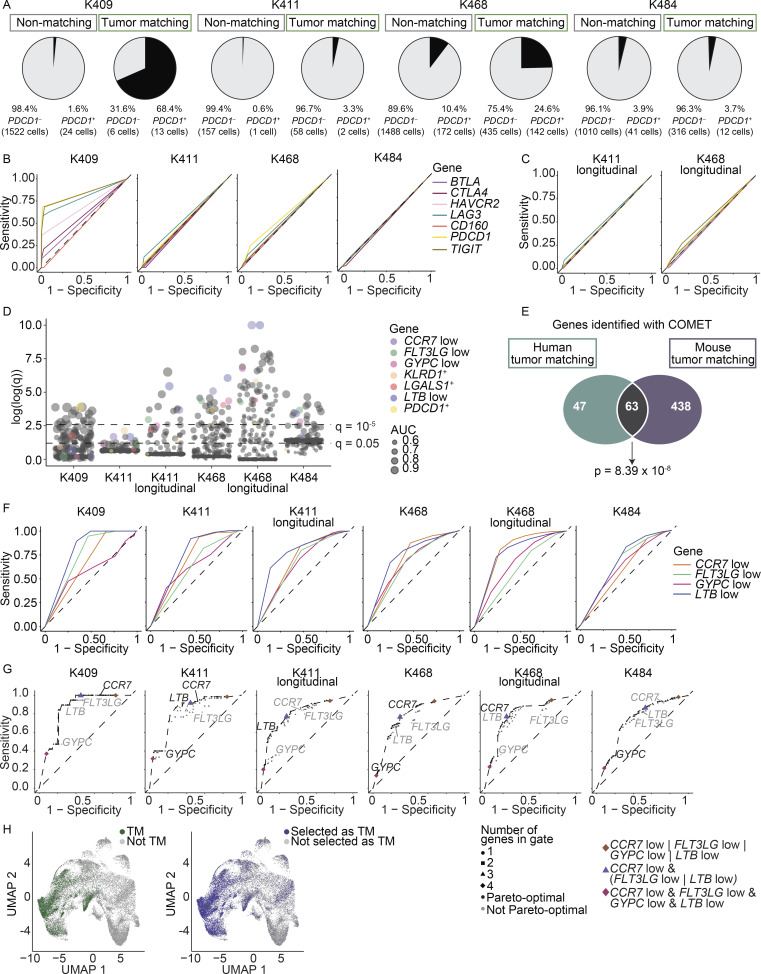
**Identification of combinations of markers for tracking TM cells across patients.** **(A)** Frequency of *PDCD1*^+^ cells (using transcript) in the initial blood sample separated by TM and non-TM cells (four experiments). **(B and C)** ROC curves showing the sensitivity and specificity of inhibitory receptor transcripts to distinguish TM cells from non-TM cells in the initial blood samples (four experiments; B) and the longitudinal blood samples (two experiments; C). Legend shared between B and C. **(D)** Plot showing the significance values from the COMET analysis across blood samples. Significance using an XL-minimal hypergeometric test with multiple hypothesis test corrections. Circles sized by AUC for sorting TM cells from non-TM cells. The y axis corresponds to the log2(*x* + 1) transformation of the −log10 of the COMET q values, capped at 10. *PDCD1* and consensus markers are highlighted with color. All other surface markers are gray. **(E)** Overlap between the single markers detected by COMET to distinguish TM cells from non-TM cells in the blood of mice (with MC38 tumors, M1–5 from three experiments) and patients (with melanoma, both treatment-naive samples and longitudinal samples, totaling six experiments). Markers included if detected as significant (q value < 0.05) in a minimum of two samples. Significance using a hypergeometric test, P = 8.39 × 10^−8^. Lists of genes and additional parameters in Table S11. **(F)** ROC curves for the consensus markers identified in D. **(G)** The sensitivity and specificity of all possible logic gates derived from combinations of genes *CCR7*^low^, *LTB*^low^, *GYPC*^low^, and *FLT3LG*^low^. Points are shaped by the number of markers used in the logical gate and colored black if they are pareto-optimal (if there is no gate with strictly better sensitivity and specificity) or gray if not pareto-optimal. A dotted line through the pareto-optimal gates represents the ROC of this combinatorial marker collection. **(F and G)** The dashed line represents the sensitivity and specificity values of random chance. **(D, F, and G)** Six experiments. **(H)** UMAP of CD8^+^ cells integrated from all patient blood samples (including longitudinal samples; data combined from six experiments). Left: True TM cells as defined by matching TCR sequence in green, nonmatching in gray. Right: Putative TM cells as determined by the best-performing gate, [*CCR7*^low^ and (*FLT3LG*^low^ or *LTB*^low^)], are colored blue; cells not expressing the marker combination in this gate in gray. For this combination, sensitivity = 0.780 and specificity = 0.716. The symbol “&” indicates the “and” gate, and the “|” indicates the “or” gate.

We next asked if cell surface markers could enrich TM cells. We first examined the use of inhibitory receptors. With the exception of patient K409, *PDCD1* RNA was detected on a minority of the TM cells (Fig. 6 A). Moreover, at the transcript level, *PDCD1* and a number of other inhibitory receptors had poor performance as predictive markers (Fig. 6, B and C; and Table S9). Our finding that the AUC values for the inhibitory receptors were hardly above chance for most patients suggested that this class of markers could not reliably enrich TM cells in blood. An independent study also found *PDCD1* to be a poor marker for cells in patient blood with TCRs matching to those in paired melanoma samples (Lucca et al., 2021).

To determine better surface markers for TM cells in humans, we again used COMET to identify transcripts that significantly enriched for TM cells (Fig. 6 D and Table S10). We observed a significant overlap between markers for the TM compartment in patient samples and the markers in mice (Fig. 6 E and Table S11), suggesting that some markers of TM cells are conserved across species and cancer types. We identified 16 near-consensus surface markers that had q < 0.05 in at least four of the six patient samples (Table S12). Of the 16 near-consensus genes, many were considered low or absent on TM cells (e.g., those for which positive expression denotes that a cell is more likely to be non-TM; see Materials and methods). The top four ranking markers based on AUC were a reduction of *LTB*,* CCR7*, *GYPC*, and *FLT3LG* on TM cells (referred to as *LTB*^low^, *CCR7*^low^, *GYPC*^low^, and *FLT3LG*^low^). Low expression of these markers is consistent with the nonnaive and/or effector or effector memory–like transcriptional state of TM cells (Fig. 4, A and C). These markers showed consensus despite differing tumor burdens and therapeutic states, showing robust AUC performance (*CCR7*^low^, 0.741; *FLT3LG*^low^, 0.620; *GYPC*^low^, 0.651; *LTB*^low^, 0.771; empirical P < 0.0001 for each; Fig. 6, D and F; Fig. S4 K; and Table S13). However, these markers featured differing strengths in sensitivity and specificity: *CCR7*^low^, 0.827 sensitivity and 0.619 specificity; *GYPC*^low^, 0.339 sensitivity and 0.819 specificity; *FLT3LG*^low^, 0.780 sensitivity and 0.447 specificity; and *LTB*^low^, 0.725 sensitivity and 0.716 specificity (empirical P < 0.0001 for each; Figs. 6 F and S4 L and Table S13). Though these top four markers are negation markers (e.g., low/negative expression on TM cells), we did observe some positive markers for TM cells lower on the list, including *KLRD1* and *LGALS1* (Fig. 6 D and Table S12), which came up in a companion study (Lucca et al., 2021).

To increase performance of surface markers to isolate TM cells from blood, we next explored the use of combinations. In all samples, marker combinations of two or more genes significantly improved performance on sensitivity and/or specificity over single markers (Fig. 6, G and H; and Table S14). The best-performing gate with even balance between sensitivity (0.780) and specificity (0.716) was [*CCR7*^low^ and (*FLT3LG*^low^ or *LTB*^low^)] (meaning that a cell has to be both low for *CCR7* and low for either *FLT3LG* or *LTB* to be classified as TM; empirical P < 0.0001 for each; Fig. 6, G and H; and Table S13). Collectively, these data highlight the utility in using combinations of markers to enrich TM cells.

**Figure S5.**
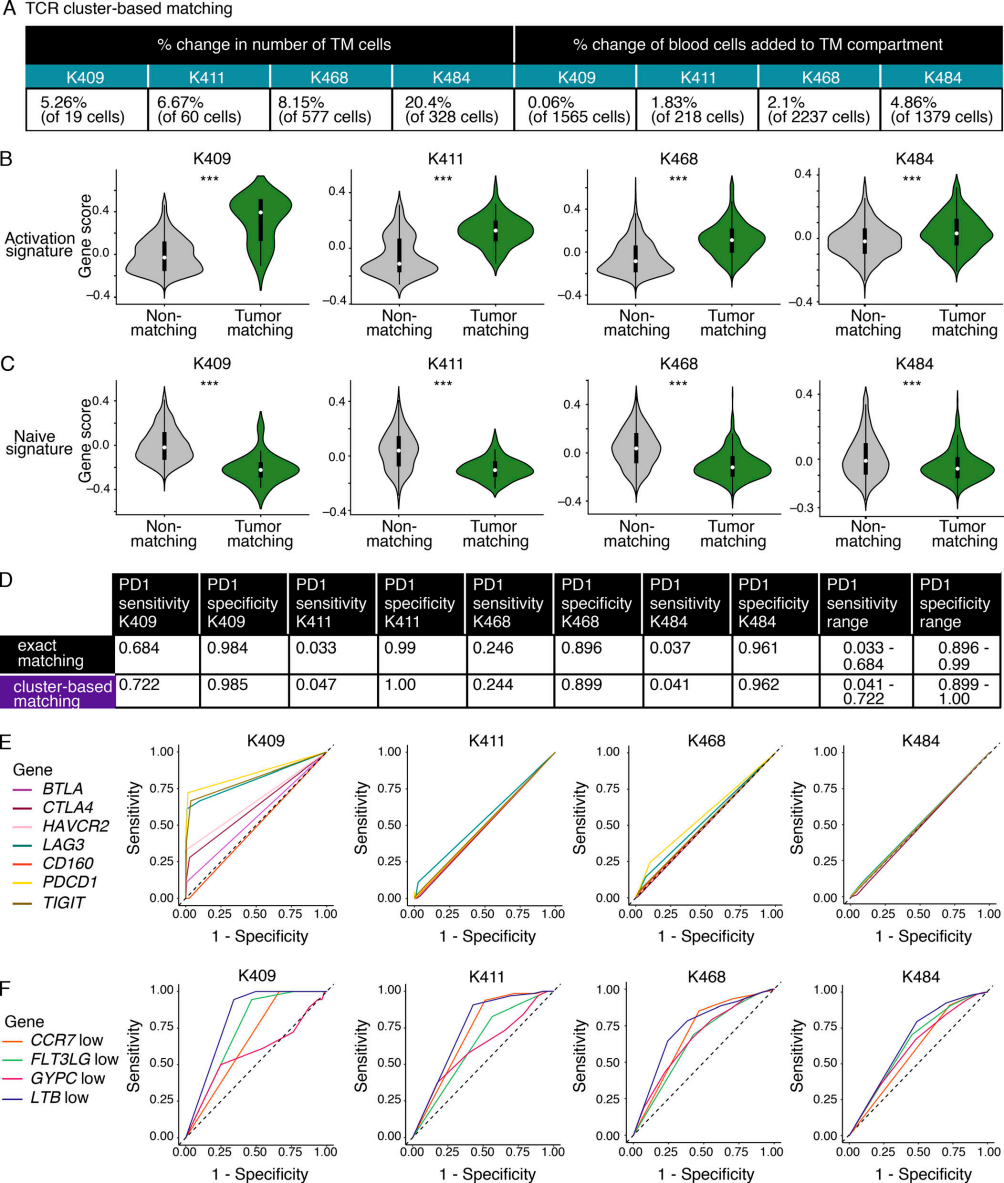
**TM CD8^+^ T cells identified using GLIPH2 and iSMART show similar signs of activation and sensitivity/specificity rates of markers as matching cells identified based on sequence matching.** **(A)** Summary metrics showing the increase in frequency of CD8^+^ T cells classified as TM cells in each of the four treatment-naive patient samples determined using the TCR cluster-based matching method (defined as cells identified as TM using both GLIPH2 and iSMART) compared with the exact sequence matching method. **(B and C)** Violin plots showing enrichment of activation (B) or naive CD8^+^ T cell signatures (C) in TM and non-TM cells on the cells identified using the TCR cluster-based matching method. Signatures derived from Akondy et al. (2017). Significance determined used Wilcoxon rank sum test. For the activation signature in B, P = 4.3 × 10^−8^ (K409), P = 8.5 × 10^−15^ (K411), P = 5.9 × 10^−99^ (K468), P = 5.4 × 10^−14^ (K484). For the naive signature in C, P = 7.2 × 10^−8^ (K409), P = 2.7 × 10^−11^ (K411), P = 2.5 × 10^−89^ (K468), P = 9.6 × 10^−12^ (K484). ***, P < 0.001. **(D)** Summary metrics showing the sensitivity and specificity of the *PDCD1* transcript to identify TM cells from non-TM cells in the blood using exact sequence matching compared with TCR cluster-based matching. **(E and F)** ROC curves for TM cells classified using the TCR cluster-based matching showing the sensitivity and specificity of a collection of inhibitory receptor genes (*PDCD1*,* BTLA*,* CTLA4*,* HAVCR2*,* LAG3*,* CD160*,**and* TIGIT*; E), or the consensus markers for identifying TM cells (*CCR7^low^*, *LTB^low^*, *GYPC^low^*, or *FLT3LG^low^*, referred to as negation markers; F), shown for each patient. Each treatment-naive patient sample was collected and run separately, totaling four independent experiments. Each patient is plotted individually.

Lastly, some TM cells may have been missed, since an exact sequence match for both the α and β chain is a highly stringent definition of a clone. To address this issue, we used two additional TCR clustering tools, GLIPH2 (Huang et al., 2020) and iSMART (Zhang et al., 2020), which increased the number of TM cells identified (5.26–20.4%; Fig. S5 A). However, TM cells were still enriched in an activation signature (Fig. S5 B), and non-TM cells were still enriched in a naive signature (Fig. S5 C). Additionally, the sensitivity of *PDCD1* and the other inhibitory receptors remained insufficient overall (Fig. S5, D and E; and Table S15). In contrast, the AUC performance of *CCR7*^low^, *FLT3LG*^low^, *GYPC*^low^, and *LTB*^low^ remained high (Fig. S5 F and Table S15). While future studies coupling larger cohorts with CITE-seq will be important to generalize findings across patients and to validate markers, the concept that marker panels could be built to monitor responses to immunotherapy in real time has tremendous clinical potential.

## Discussion

There is significant interest in monitoring anti-tumor immune responses. The blood is a conduit of immune cell trafficking, making it a window into these responses. However, comprehensive profiling of tumor antigen–specific T cells in the blood has been challenging. Use of the TCR as a molecular barcode to track TM cells provides an effective way to enrich tumor-relevant populations. This approach is potentially less biased than alternatives like PD-1 expression, while capturing a larger breadth of the anti-tumor response than individual peptide/MHC tetramers.

There are technical and biological considerations with this method. First, paired blood and tumor samples are required to identify TM cells. Second, sampling depth in the tumor may impact the proportion of the TM repertoire detected. However, the TM cells detected here showed consistent transcriptional states and markers for their isolation despite variability in the depth of coverage across patients. Third, use of negation markers can be challenging in single-cell data, since these datasets contain a large number of zero values, and it is debated whether counts of zero are due to true biology or technical artifacts. It is generally accepted that genes receiving zero counts are either not expressed or expressed to a low level within a cell (Choi et al., 2020), and work has concluded that the zero measurements in count data reflect true biology (Choi et al., 2020; Hafemeister and Satija, 2019; Svensson, 2020; Townes et al., 2019). We therefore conclude that TM cells are lower for *GYPC*,* CCR7*,* LTB*,**and* FLT3LG* than non-TM cells. Fourth, bystander T cells specific for pathogens have been identified in mouse and human tumors (Mognol et al., 2017; Simoni et al., 2018). Further work is needed to determine if the cells with the bystander transcriptional signature indeed have TCRs that are specific only to pathogens and not tumor. Follow-up studies aimed at developing panels that can distinguish between tumor-specific and bystander cells would be useful.

The majority of TM cells in the blood of our advanced melanoma patients displayed an effector and/or effector memory–like phenotype. This was counter to our predictions in that we expected a more exhausted-like profile. While patient K409 showed similar enrichments for an exhaustion signature between matching clones in blood and tumor, the other three patients showed elevated exhaustion scores in the tumor. This finding highlights the importance of using the TCR rather than surrogate markers such as PD-1, which make an assumption about expected differentiation states of relevant cells. As a class, inhibitory receptor transcripts performed poorly at distinguishing TM cells in the blood, with the exception of patient K409, suggesting that there may be better cell surface markers for identifying tumor-relevant cells in blood.

Three markers validated here in mice for identifying TM cells were NKG2D, CD39, and CX3CR1. When comparing effector, memory, and exhausted populations, *Klrk1* shows the highest expression in memory CD8^+^ T cells (assessed from GEO accession no. GSE41867; Doering et al., 2012), and NKG2D is important for optimal memory formation (André et al., 2012; Ferrari de Andrade et al., 2018; Prajapati et al., 2018; Wensveen et al., 2013; Zloza et al., 2012). CD39 is associated with exhaustion (Gupta et al., 2015). CX3CR1 correlates with effector CD8^+^ T cell differentiation, with the highest levels on the most effector-like cells (Gerlach et al., 2016). How NKG2D, CD39, CX3CR1, and other candidate markers impact the function of TM cells remains to be determined. Some of the markers identified may be specific to this tumor type or its location in the skin and may differ with tumor type or location. However, a number were associated with a general program of trafficking to inflamed tissues and were not skin specific, including *Ccr2*,* Ccr5*,* Cx3cr1*,* Itga4*,* Itgb1*, and *Itgb2* (Liu et al., 2006; Masopust et al., 2010). The significant overlap between TM markers in the mouse MC38 model and melanoma patients suggests that there can be similarities that span tumor type and species.

In mice, the TM population in the blood was fairly homogenous. However, blood-matching clones in tumors showed significant transcriptional diversity. These data suggest that TM cells have a high degree of plasticity upon entering the tumor, and the tumor microenvironment influences the development of diverse functional states. On a clonal basis, TM cells in the blood were less exhausted than their blood-matching counterparts in tumor, in both mice and patients, with the exception of K409. In the two longitudinal patient samples, the clones detected in the second blood sample were more dysfunctional than the first, consistent with the notion that exhaustion continues to develop over time (Wherry and Kurachi, 2015). However, clones in blood appeared less exhausted than clones in tumor, suggesting that blood may be a reservoir of less dysfunctional cells.

In summary, we identified CD8^+^ T cells in blood that had matching TCRs with CD8^+^ T cells in mouse or human tumors. TM cells in blood were generally less dysfunctional than matching clones in tumor. Additionally, we provide evidence for an exciting and tractable innovation: the use of combinatorial marker panels to isolate TM cells in blood. These panels were consistent over time, across patients, and robust to sampling variation. Follow-up studies interrogating how immunotherapies such as PD-1 blockade impact TM cells will be highly relevant to determining predictors of response versus resistance. Our algorithmic approach to generate marker panels to identify TM cells coupled with future longitudinal studies could assist with creation of diagnostics, potentially allowing monitoring of the anti-tumor immune response in real time without the need for single-cell sequencing.

## Materials and methods

### Experimental model and subject details

#### Mice and cell lines

WT female C57BL/6 mice were purchased from The Jackson Laboratory (stock number 000664). Tumor cells were implanted into mice at 8–10 wk of age. Mice were maintained at Harvard Medical School in specific pathogen–free facilities under standard housing, husbandry, and diet conditions in accordance with Institutional Animal Care and Use Committee (IACUC) and National Institutes of Health guidelines. All experimental procedures performed were approved by the IACUC at Harvard Medical School.

For tumor studies, MC38 colon adenocarcinoma cells (a gift from Dario Vignali, University of Pittsburgh School of Medicine, Pittsburgh, PA) were used. MC38 cells were grown in DMEM supplemented with 10% FBS, 100 U penicillin, and 100 µg streptomycin in a 37°C incubator with 5% CO_2_. Cells were harvested at passage 2–3 after thaw, and 2.5 × 10^5^ tumor cells were injected subcutaneously into the flank of mice anesthetized with 2.5% 2,2,2,-tribromoethanol (Avertin). Tumors were measured every 2–3 d using calipers, and mice were sacrificed when tumors reached a 2-cm^3^ volume, ulceration, or a body condition of >2 in accordance with IACUC guidelines. Tumor volume was determined using the formula for the volume of an ellipsoid, 1/2 × *D* × *d*^2^, where *D* is the major axis of the tumor and *d* is the minor axis. Tumors were harvested from mice at days 18–23 after implantation for scRNAseq and flow validation experiments as indicated in the figure legends.

#### Clinical samples

Studies of patients with melanoma were approved by the Committee on Human Research from the Human Research Protection Program at University of California, San Francisco (UCSF; CC138510) and by the Institutional Review Board of UCSF under protocol 13-12246. All patients provided written, informed consent before biopsy and/or blood collection. Patient sample details including location of biopsy, treatments following initial blood/tumor sampling, gender, age, and timing of longitudinal blood collection can be found in Fig. S4, A and B, and Table S7.

### Method details

#### Lymphocyte isolation from mouse tissues

Peripheral blood was collected from mice using the retroorbital bleeding route, and blood was collected into 4% sodium citrate (Sigma-Aldrich) to prevent clotting. RPMI + 10% FBS was added to dilute out the anti-coagulant, and then white blood cells were separated from red blood cells using centrifugation through histopaque-1083 (Sigma-Aldrich). The white blood cell layer at the interface between the histopaque and remaining medium was subsequently subjected to staining for flow cytometry analysis or sorting for scRNAseq.

Tumors were dissected and mechanically disaggregated. For flow cytometry validations, a GentleMACS (Miltenyi) was used for disaggregation, whereas for scRNAseq scissors were used to finely mince the tumors instead of the GentleMACS. The dissociated tissue was digested with collagenase type I (400 U/ml; Worthington Biochemical) for 20–30 min at 37°C. Samples were then passed through a 70-µm filter, and lymphocytes were enriched using centrifugation through a Percoll gradient (40% and 70%). The enriched lymphocyte layer at the 40%/70% interface was subsequently stained for flow cytometry or sorted for scRNAseq.

#### Flow cytometry and sorting of mouse samples

Single-cell suspensions were generated as described above. Suspensions were labeled with LIVE/DEAD Fixable Near-IR Cell Stain in PBS (Thermo Fisher Scientific) to exclude dead cells from downstream analyses. Cells were preincubated with TruStain Fc Receptor Block (anti-mouse CD16/CD32, clone 93; BioLegend), and then labeled with extracellular antibodies including CD3 (clone 145-2C11) and CD8α (clone 53-6.7; from BD); CD11a (clone M17/4; from Thermo Fisher Scientific); CCR2 and NKG2I (from R&D Systems); Lag3 (clone C9B7W; from Bio-Rad); and CD45.2 (clone 104), PD-1 (clone RMPI-30), CX3CR1 (clone SA011F11), CD62L (MEL-14), CD44 (IM7), CCR5 (clone HM-CCR5), CXCR6 (clone SA051D1), CD49D (clone R1-2), CD18 (clone M18/2), CD29 (clone HMβ 1-1), CD48 (clone HM48-1), CD94 (clone 18d3), NKG2D (clone CX5 or C7), CD39 (clone Duha59), NKG2A (clone 16A11), NK1.1 (clone PK136), Tim-3 (clone RMT3-23), CD160 (clone 7H1), Slamf7 (clone 4G2), TIGIT (clone IG9), and NRP1 (clone 3E12; from BioLegend). Flow cytometry labeling (without inclusion of Feature Barcoding antibodies from BioLegend) was performed in PBS supplemented with 2% FBS. For CITE-seq validation experiments, cells were labeled with TotalSeqC antibodies against CD39 (TotalSeq C0834, clone Duha59) and CX3CR1 (TotalSeq C0563, clone SA011F11) as directly conjugated antibodies and NKG2D as a biotin/streptavidin reaction (NKG2D-biotin clone C7 paired with TotalSeq C0971-Steptavidin; from BioLegend). Labeling with Feature Barcoding antibodies was performed in PBS supplemented with 2% BSA and 0.01% Tween. Samples were acquired on a FACSymphony (BD Biosciences) and analyzed with Flow Jo software (BD Biosciences). Flow cytometry–based sorting for scRNAseq was performed using a FACSAria (BD Biosciences). Because we expected TM cells in the blood to be rare, we sorted for CD44^+^ CD8^+^ T cells to enrich for antigen-experienced populations in the blood (full sorting strategy = live, CD45.2^+^, CD3^+^, CD8α^+^, CD44^mid-high^). Although all CD8^+^ T cells sorted from blood expressed some level of CD44, cells from mouse 1 (M1, experiment 1) were sorted on CD44^high^, while cells from mouse 2 and mouse 3 (M2 and M3, experiment 2) included both CD44^mid^ and CD44^high^ cells. Tumor samples were sorted based on live, CD45.2^+^, CD3^+^, CD8α^+^.

#### scRNAseq of mouse samples

Gene expression and TCR libraries for mouse samples were generated using the Chromium Single Cell 5′ Library and V(D)J Reagent Kit (10X Genomics) according to the manufacturer’s recommendations. For samples requiring Feature Barcoding libraries to detect TotalSeqC antibodies (from BioLegend), the Chromium Single Cell 5′ Feature Barcode Library Kit (10X Genomics) was used according to the manufacturer’s recommendations. Following sorting as described above, ~10,000 cells per sample were loaded into each channel of the Chromium Chip, and recommendations were followed assuming targeted cell recovery of 2,001–6,000 cells. Libraries were sequenced on a NextSeq sequencer (Illumina) by the Dana-Farber Cancer Institute Sequencing Core. Gene expression libraries and Feature Barcoding libraries were sequenced using the 26 × 8 × 91-bp parameters recommended by 10X Genomics. TCR libraries were sequenced using the 150 × 8 × 150-bp parameters recommended by 10X Genomics. Based on approximate cell numbers expected, we sequenced a minimum of 20,000 reads per cell for gene expression libraries and 5,000 reads per cell for TCR and Feature Barcoding libraries.

#### Lymphocyte isolation from human tissue samples

Human melanoma tumor samples were mechanically dissociated and enzymatically digested overnight for 12–14 h. Following fine mincing with scissors, samples were digested in RPMI media (Gibco) containing 250 U/ml type IV collagenase (4188; Worthington Biochemical Corp.), 20 µg/ml DNase (SDN25-1G; Sigma-Aldrich), 10% FBS (Alphabioregen), 1% Hepes (Gibco), 1% penicillin/streptomycin (Gibco), and 2 mM glutamine (GLUTAmax; Gibco) at 37°C in a tissue culture incubator with 5% CO_2_. Following overnight incubation, digestion was quenched with excess media, and samples were transferred to 50-ml conical tubes, briefly shaken, and filtered through a 100-µm sieve. Samples were pelleted and washed in media before downstream applications.

#### Lymphocyte isolation from human blood samples

Blood from patients with melanoma was collected in heparinized or EDTA tubes and diluted with an equal volume of PBS before being layered over a Ficoll Paque PLUS gradient (GE Healthcare) in 50-ml conical tubes that were centrifuged at room temperature for 15 min at 932 *g*. Cells were isolated from the Ficoll/PBS interface and washed at least twice in PBS/2% FBS before downstream applications. For the two patients with longitudinal blood samples processed (K411 and K468), both patients still had tumor at the time of longitudinal blood collection.

#### Flow cytometry and sorting of human samples

Melanoma tumors (primary tumors or metastases as indicated in Fig. S4 B and Table S7) or blood were stained in PBS with Tonbo Ghost Dye Violet 510, anti-CD45 (clone H130), anti-CD3 (clone SK7), anti-CD4 (clone SK3), and anti-CD8 (clone SK1). Some samples were additionally stained with anti-PD-1 (clone EH12.2H7), anti-CD25 (clone M-A251), anti-CD27 (clone LG.7F9), and anti-CD127 (clone HIL-7R-M21). CD8^+^ T cells were sort purified as singlet, live, CD45^+^, CD3^+^, CD4^−^, CD8^+^ events on an Aria 2 or Aria 3u (BD) in the UCSF Parnassus Flow Cytometry Core. In some cases the total CD3^+^ T cell population was sort purified as singlet, live, CD45^+^, CD3^+^ events, and CD8^+^ T cells were identified bioinformatically. Cells were counted after sorting on a hematocytometer and resuspended to target ~1,000 cells/µl in media with 10% FBS for scRNAseq.

#### scRNAseq of human samples

Following sorting, cells were prepared for scRNAseq using the 10X Chromium Platform (10X Genomics) by the Institute for Human Genetics at UCSF. Cells were processed following the recommended protocol with the Chromium Single Cell 5′ Library Construction kit and Chromium Single Cell V(D)J Enrichment Kit (Human T Cell; Single Cell 5′ PE Chemistry). Libraries were run on a HiSeq 4000. FASTQ files were generated and analyzed with Cell Ranger (version 3.0.2) by the UCSF 10X Genomics Core using the GRCh38 human reference genome for alignment.

#### Demultiplexing and read processing

Raw reads were processed using Cell Ranger version 3.0.2 to generate raw counts matrices of gene expression and CSV files corresponding to TCR clonality. The GRCh38 human reference genome was used for alignment of human samples, and the mm10 mouse reference genome was used for alignment of mouse samples. Aether version 1.0 (Luber et al., 2018) was used to process certain resource-heavy jobs on compute instances rented from Amazon Web Services.

#### Computational processing of gene expression data

All analyses were conducted using R version 3.6.1 and Seurat version 3 with additional utilization of the dplyr, data.table, ggplot2, cowplot, viridis, gridExtra, RColorBrewer, ggpubr, ggrepel, gtools, DescTools, doParallel, doSNOW, and tibble packages. Seurat objects were created with the min.cells parameter set to 3 and the min.features parameter set to 400. Filtering cells based on expression of housekeeping genes was conducted using the human and mouse (where appropriate) gene lists maintained by the Seurat developers (available on the Satija laboratory website), with cells passing the filtering criteria if they had expression >0 for more than half of the genes in the list. Subsequently, the MitoCarta database from the Broad Institute was used to filter out cells based on expression of mitochondrial genes (Calvo et al., 2016). Cells were filtered out if they expressed >500 of the 1,158 mitochondrial genes in humans, or if the number of mitochondrial genes expressed was more than two SDs from the mean in mouse.

Data were normalized using the default Seurat function (generating log-transformed transcripts-per-10K read measurements) followed by scaling, and variable genes were found using “ExpMean” for the mean.function parameter and “LogVMR” for the dispersion.function parameter. The RunPCA function was run using 50 principal components, and then the FindNeighbors function was run using 30 dimensions. Subsequently, the FindClusters function was run with a resolution aiming to generate five to seven biologically meaningful clusters per sample. To filter for CD8^+^ T cells in humans, clusters were kept if (a) the proportion of cells in the cluster with at least two genes out of *CD3E*, *CD3D*, or *CD3G* being expressed was >30% and either (b) *CD8B* was expressed in >30% of cells in the cluster, *CD8A* was expressed in >30% of cells in the cluster, *FOXP3* was expressed in <5% of cells in the cluster, and *CD4* was expressed in <5% of cells in the cluster or (c) *MKI67* was expressed in >70% of cells in the cluster and either *CD8A* or *CD8B* was expressed in >20% of the cells in the cluster. This last criterion was to account for proliferating clusters. In mice, which had less contamination from non-CD8^+^ T cells due to prior sorting, clusters were kept if >30% of cells in the cluster expressed *Cd3e*, *Cd3d*, or *Cd3g* and if >30% of cells in the cluster expressed *Cd3e* and *Cd8a* while having <5% of the cells express *Foxp3*. When applicable, samples were integrated using the SCTransform method (Hafemeister and Satija, 2019).

The samples from the first three mice with MC38 tumors were integrated to generate an “integrated blood” sample and an “integrated MC38 tumor” sample as a discovery cohort. These three biological replicates were generated between two independent experiments (M1, experiment 1; M2 and M3, experiment 2). M4 and M5 (two biological replicates) were generated as a separate validation cohort from one experiment that included CITE-seq. For patient samples, each patient was collected and processed separately, making each patient an independent experiment. Therefore, we have four independent experiments for treatment-naive patients and two independent experiments for longitudinal follow-up analyses. The majority of analyses were performed on each patient individually, not the integrated sample. Integration was performed for clustering and uniform manifold approximation and projection (UMAP) visualization purposes and included only the initial four pretreatment samples with the exception of Figs. 6 H and S4 L, which included all six samples (the initial four pretreatment samples and the two longitudinal samples). For patient K409, tissue from both the primary tumor site and an involved LN was processed for scRNAseq. For this patient, the data for primary tumor and the involved LN were pooled, and cells in the blood were considered TM if they had a TCR sequence matching either tumor resection.

Upon obtaining transcriptional clusters in the integrated datasets, up-regulated genes associated with each cluster were determined via the Wilcox rank sum test implemented in the FindAllMarkers function in Seurat. Cells were classified as positive for the PD-1 transcript (*Pdcd1* in mice, *PDCD1* in humans) if they had any number of reads >0. To classify mouse cells as positive for the *Klrk1* (encoding NKG2D), *Entpd1* (encoding CD39), and/or *Cx3cr1* (encoding CX3CR1), a more stringent cutoff was used for a cell to qualify as positive, determined by COMET (Delaney et al., 2019).

For enrichment analysis tests (Fig. 1 E), all genes were ranked by their P value and fold change, and then the two ranking values were aggregated to create a single ranking by taking the mean of the P value and fold change rankings. We then searched for significant associations with gene signatures by using the ranked list in the PreRanked analysis of gene set enrichment analysis (Subramanian et al., 2005). Default settings were used, except permutations was set to 100, the enrichment statistic was set to “classic,” and the max size was set to 2,500. The signature sets used were all gene ontology terms, Kegg and reactome pathways, and immune signatures from MSigDB (groups c2, c5, and c7). Gene signatures derived from the literature were also analyzed as cited in the figures and text.

To perform the clonal-corrected DE gene analysis comparing TM cells in the blood to blood-matching cells in the tumor (Table S6), the nonnormalized integrated mouse blood object was subsetted to keep only TM cells, which were then collapsed into their clones such that for each gene, the counts for all the cells in a clone were summed together. This was done for the integrated tumor as well, but with blood-matching cells. The tumor and blood-derived datasets were then merged to a single object, and the edgeR package was used to call differential expression. Genes were considered if they expressed at least one count per million, and then counts were normalized using the trimmed mean of M values. Taking into account the paired nature of matching clones in blood and tumor, genes were fitted to a generalized linear model using the ‘glmFit’ function, and likelihood ratio tests were conducted to detect DE genes between blood and tumor with the ‘glmLRT’ function.

#### Single-cell TCR and clonal analysis

Cells for which at least one α and one β chain were annotated in the TCR data were determined as matching or nonmatching based on whether there was a cell in the paired tissue data that had the exact same α and β chain composition as the given cell. Only cells that had at least one α chain and one β chain annotated were included in all of the analyses comparing matching to nonmatching cells. Two cells were assigned to be in the same clone if they had both the exact same α and β chains assigned based on the amino acid sequence. If cells had more than one α and β chain, they were considered matching if all of the α and β chains detected were shared. This strict definition was used to ensure that each pair of cells within the same clone had complete similarity of the TCR chains detected, and hence was with high probability derived from the same T cell clone. TCR information was also used to quantify clonal expansion. The extent of clonal expansion was determined by counting the number of cells in each clonotype.

To define TM status by clustering of TCRs, two algorithms were employed: GLIPH2 (Huang et al., 2020) and iSMART (Zhang et al., 2020). For each patient, the joint collection of blood and tumor CD8^+^ TCRs were submitted to each algorithm individually for clustering on default parameters. All resultant clusters that included at least one TCR found in the tumor sample were considered to represent reactivity to a tumor antigen, and therefore all blood CD8^+^ T cells with TCRs belonging to these clusters were considered TM cells. In general, the results of GLIPH2 and iSMART were concordant with 4,926 cell TM labels in agreement and 70 in disagreement. To buffer this analysis against variation in algorithm and parameter choices, we disregarded the 70 cells for which the two algorithms gave conflicting results (<1.43% of cells).

#### Functional annotations of Seurat clusters

Functional annotations for Seurat clusters were manually curated using a combination of up-regulated genes for each cluster (Table S1 for mouse and Table S8 for human) and visual inspection of key markers using UMAP visualization. Key markers used for aiding in annotation included *Sell*,* Tcf7*,* Lef1*,* Ccr7*,* Il7r*,* S1pr1*,* Klf2*,* Cxcr3*,* Klrg1*,* Cx3cr1*,* S1pr5*,* Tnf*,* Ifng*,* Il2ra*,* Gzmb*,* Prf1*,* Mki67*,* Slamf6*,* Pdcd1*,* Lag3*,* Tigit*,* Cd160*,* Havcr2*,* Ctla4*,* Bst2*,* Irf1*,* Irf2*,* Irf7*,* Mx1*,* Ccr6*,* Rorc*,* Cxcr6*,* Itgae*,* cd69*,* Tbx21*, and *Eomes.* Transcriptional signatures in blood were consistent with naive, central memory, effector, and effector memory cells, and signatures in tumor were consistent with diverse exhausted subsets, effector-like, resident memory–like, naive/central memory–like, IFN-stimulated, and cycling populations, as previously reported (Best et al., 2013; Guo et al., 2018; He et al., 2016; Im et al., 2016; Kakaradov et al., 2017; Kurtulus et al., 2019; Miller et al., 2019; Milner et al., 2017; Sade-Feldman et al., 2018; Siddiqui et al., 2019; Tirosh et al., 2016; van der Leun et al., 2020; Yost et al., 2019).

Clusters that expressed high levels of *Sell*,* Tcf7*,* Ccr7*,* Il7r*,* S1pr1*, and *Klf2* and lower levels of *Klrg1*,* Cx3cr1*,* S1pr5*,* Tnf*,* Ifng*,* Gzmb*,* Prf1*,* Mki67*, and the inhibitory receptors (e.g., *Pdcd1*,* Havcr2*, and* Ctla4*) were considered naive and/or central memory–like. Clusters that expressed high levels of *Klrg1*,* Cx3cr1*,* S1pr5*,* Tnf*,* Ifng*,* Gzmb*, and *Prf1* and low levels of *Sell*,* Tcf7*,* Lef1*,* Ccr7*, and *Il7r* were considered effector and/or effector memory–like. Exhausted subsets were classified as those expressing multiple inhibitory receptors (*Pdcd1*,* Havcr2*,* Lag3*,* Tigit*, etc.), low levels of naive and/or central memory–like markers, and generally lower levels of some effector molecules such as *Klrg1* and *Cx3cr1*. The exhausted populations were further subdivided into progenitor-like (based on expression of *Tcf7* and lower levels of *Havcr2*), intermediate-like (based on low levels of *Tcf7* and *Havcr2* and expression of other IRs, including *Pdcd1*,* Ctla4*,* Lag3*,* CD160*, etc.), and terminal-like (based on high levels of multiple inhibitory receptors, including *Pdcd1*,* Havcr2*,* Ctla4*,* Lag3*,* Cd160*, etc.). An IFN-stimulated cluster was defined based on over representation of IFN responsive genes in the up-regulated gene list, including *Bst2*,* Irf1*,* Irf2*,* Irf7*,* Stat1*,* Stat2*, and *Mx1.* Clusters containing cells that were undergoing cell cycle were identified based on over representation of cell cycle genes (including *Mki67* and several *Kif*,* Cdk*,**and* Cdc* genes). Lastly, resident memory–like populations were identified based on expression of *Itgae*, *Itga1*, and *Cxcr6*.

#### Transcriptional signature analysis

We computed the extent to which gene signatures were expressed in cells by using Scanpy’s ‘score_genes’ function on the centered and scaled gene count data objects (Wolf et al., 2018). Because gene signature computation is relative (following centering and scaling of the gene expression data), data of all cells compared were merged before the centering and scaling procedure. Violin plots were generated with the ‘seaborn’ package in Python. Signatures were derived or obtained from previously published datasets. For mouse, the naive signature was from Kaech et al. (2002), the CD8 T cell activation signature was from Sarkar et al. (2007), the cell cycle signature was from Kowalczyk et al. (2015), the TRM signature was from Beura et al. (2018), the bystander signature was from Mognol et al. (2017), and the effector-like and terminally exhausted signatures were from Miller et al. (2019). For human, the naive and activation signatures were derived from Akondy et al. (2017), and the exhaustion signature was obtained from Sade-Feldman et al. (2018).

To create the plot shown in Fig. S3 C, all cells were merged into a single data object and normalized to 10,000 counts per cell. Only cells with at least one α and one β chain were included. Then each count was logarithmized according to log(1 + *X*), where *X* is the gene count, and each gene was standardized to unit variance and zero mean. Given a signature, a score was calculated for each cell with Scanpy’s ‘score_genes’ function. The average of the cell scores was calculated for each sample.

#### Machine learning

Classification of TM cells in mouse (Fig. 2, A and B) was conducted using L2 regularized logistic regression using the Scikit-learn package in Python version 2.7 (Pedregosa et al., 2011). Plots were generated using matplotlib. For the logistic regression, the liblinear solver was used with an l2 penalty and C parameter set to 0.02.

#### Calculation of AUC

For each gene in each patient, the AUC in distinguishing TM cells from non-TM cells was computed with the AUC( ) function of the R DescTools package. To construct an input for the AUC( ) function, we calculated a vector of (1 − specificity) values and a vector of corresponding sensitivity values from 39 potential expression level thresholds for dividing the two populations. For each gene in each patient, the 39 thresholds were every fifth percentile expression of the gene (21 values including 0th percentile and 100th percentile) combined with 18 evenly spaced expression values between the minimum and maximum, to account for heavily skewed distributions in which useful thresholds may lie above the 95th or below the 5th percentile. To these input vectors we added (0,0) and (1,1), representing the trivial options of selecting none and all of the cells as TM, respectively.

#### Similarity of TM component across patients

Similarity between samples in terms of the power of a transcript in distinguishing the TM component from the non-TM component was computed in Fig. 5 C via pairwise correlation of gene transcript AUC values for selecting the TM component. The AUC for each gene transcript in each patient was calculated as described above, and all pairwise combinations between patient samples were plotted for each gene, resulting in $\left(\begin{array}{c} 6\\ 2 \end{array}\right)$ = 15 points per gene. Negation markers are represented by AUC values <0.5 when selecting the TM component. To mitigate the x axis being arbitrarily biased toward the patients appearing first in the data, x and y coordinates were switched for each point with a probability of 0.5 and a random seed set to 27 in R. With the function stat_cor( ) from R package ggpubr, Pearson correlation statistics were computed for the resultant x and y values, stratified by whether each sample pair was within the same patient or across different patients. The plot is restricted to transcripts that were measured in all six patient samples.

#### COMET

COMET (Delaney et al., 2019) runs were conducted with version 0.1.12 and the default *X* parameter (0.15) and with the L parameter set to the minimum of (1) 10 × *K* and (2) 0.35 × *N*, where *K* is the number of TM cells and *N* is the total number of cells with at least one α and one β chain annotated, to account for our willingness to allow for greater levels of contamination in the identified TM samples than allowed by default.

The full lists of unranked markers from COMET are provided in Table S4 for mouse and Table S10 for human. In these files, the COMET-determined threshold value (column labeled “cutoff_val”) is indicated for each marker when used as a positive marker or a negation marker. Negation markers are labeled as “marker_negation,” whereas positive markers are listed as simply “marker.” For positive markers, a cell is predicted to be TM if its gene expression is above the threshold. For negation markers, a cell is predicted to be TM if its gene expression is lower than the threshold. In COMET’s original output, negation markers are multiplied by (−1). We therefore took the absolute value of all reported thresholds in the output tables to increase clarity. Since “negation” does not necessarily equate to no expression for a marker, throughout, the text cells deemed positive for a “negation marker” are referred to as “marker low” instead of “marker negative.”

#### Ranking singleton human markers

Leading candidate markers for follow-up analysis from the human samples were determined by the number of patient samples in which a given marker reached significance in COMET (q < 0.05). From the input list (Chihara et al., 2018), we removed *CD8A* because this is a lineage-defining marker and therefore not ideal for separating the TM and non-TM components, along with cytokines *CCL4*, *CCL5*, and *MIF* in order to strictly consider surface-expressed markers. The 16 markers derived from this filtered list had q < 0.05 in the majority of patient samples (four of six) and were considered for follow-up analysis. These 16 candidate markers were ranked in order of descending average AUC in distinguishing TM cells from non-TM cells across patient samples (Table S12). The top four on this list were negation markers for *LTB*,* CCR7*,* GYPC*, and *FLT3LG*, meaning that low or absent expression of these markers is associated with TM cells.

#### Empirical P values and confidence intervals for gate performance

Confidence intervals for gate AUC, sensitivity, and specificity were determined by 10,000 iterations of random bootstrap resampling with replacement in the pooled CD8^+^ blood cell population and separately with respect to each individual patient blood sample. The 95% confidence intervals go from the 2.5th percentile to the 97.5th percentile of 10,000 bootstrapped recalculations of AUC, sensitivity, and specificity. A null distribution for each gate was generated iteratively through each resample by permuting the TM labels and calculating the AUC, sensitivity, and specificity from the resultant datasets. These distributions represented the null hypothesis that each given marker was sorting TM cells from non-TM cells by chance alone. Reported empirical P values <0.0001 reflect the observation that the point estimate for the marker’s AUC, sensitivity, or specificity was never observed in the 10,000 iterations of the null.

#### Ranking of combinatorial marker gates

All possible one-, two-, three-, and four-gene logical gates were enumerated from the four top-ranking markers in the patient samples (*CCR7*^low^, *FLT3LG*^low^, *GYPC*^low^, and *LTB*^low^) and evaluated for their sensitivity (e.g., capture rate) and specificity (e.g., contamination rate) in isolating the TM component in each patient at a universal threshold of 0.001 unique molecular identifiers. The optimal threshold to discriminate these two populations must be calibrated to the distribution of read counts as well as the target sensitivity and specificity. We used COMET to determine the optimal threshold for each marker in each patient sample (Table S14) and chose a universal threshold of 0.001 following manual inspection (COMET-derived thresholds averaged across patient samples were 0.001, 0.001, 0.833, and 0.334 for *CCR7*^low^, *FLT3LG*^low^, *GYPC*^low^, and *LTB*^low^, respectively, and any threshold between 0 and 1 is functionally equivalent when applied to count data). To identify the best-performing combinatorial gate, we computed a penalty for each gate based on both its distance from perfect sensitivity and specificity and its balance between the two metrics. To calculate this penalty, we first computed the Euclidean distance from perfect sensitivity and specificity (corresponding to (0,1) on ROC curve plots) to the point on the plot representing that gate’s sensitivity and specificity in the pool of CD8^+^ cells across all patient blood samples. To this Euclidean distance, we added the difference between the gate’s sensitivity and specificity in the pool of CD8^+^ T cells across all patient blood samples in order to promote the selection of the most balanced gate. This process identified [*CCR7*^low^ and (*FLT3LG*^low^ or *LTB*^low^)] as the best-performing and most balanced gate (lowest penalty).

### Quantification and statistical analysis

#### Flow cytometry validations in mouse

Statistical analyses for flow cytometry data were performed with Prism software (GraphPad), and P values <0.05 were considered statistically significant. Multiple *t* tests were performed using the Holm–Sidak method with α = 0.05. Each row was analyzed individually, without assuming a consistent SD. Asterisks indicating significance in the figures correspond to P < 0.05 (*), P < 0.01 (**), and P < 0.001 (***). Statistical tests used for computational analyses are indicated in the corresponding figure legends and Materials and methods sections. Exact P values for significant comparisons are indicated in the figure legends and supplemental tables.

### Resource availability

Further information and requests for resources and reagents should be directed to and will be fulfilled by the corresponding authors. This study did not generate new unique reagents. The gene expression scRNAseq data for patients K409 and K411 (initial blood/tumor pair) and the TCR data (for tumor) can be found on GEO with accession no. GSE148190 (Mahuron et al., 2020). The scRNAseq generated during this study can be found on GEO as a SuperSeries with accession no. GSE159252. Within the SuperSeries, the mouse scRNAseq data can be with accession no. GSE158520, and the human scRNAseq data can be found with accession no. GSE159251. Code used for this study is available on GitHub at https://github.com/MSingerLab/Blood_Tumor_Code.

### Online supplemental material

Fig. S1 is associated with Fig. 1 and provides additional details characterizing the scRNAseq discovery cohort in mice. Fig. S2 is associated with Fig. 2 and provides additional information about the COMET output, flow cytometry validations in mice, and combinations of NKG2D, CX3CR1, and CD39 in our flow cytometry and CITE-seq experiments. Fig. S3 is associated with Fig. 3 and provides additional comparisons between matching clones in blood and tumor in mice. Fig. S4 is associated with Figs. 4 and 6 and provides supporting information regarding the scRNAseq in the melanoma patients. Fig. S5 is associated with Figs. 4 and 6 and details results from alternative methods to identify matching clones based on the TCR in melanoma patients. Table S1 shows up-regulated genes for each Seurat cluster in mouse integrated blood and MC38 tumor samples. Table S2 shows up-regulated genes for TM and non-TM CD8+ T cells in the peripheral blood of mice with MC38 tumors. Table S3 is a full list of pathways and signatures enriched in TM and in non-TM CD8^+^ T cells from the peripheral blood of mice with MC38 tumors. Table S4 shows significance measures calculated with COMET in sorting TM from non-TM CD8^+^ T cells in the blood of mice with MC38 tumors. Table S5 shows the sensitivity and specificity of all possible gates made from combinations of NKG2D, CD39, and CX3CR1, measured by CITE seq in mice. Table S6 lists DE genes between TM cells in blood and blood-matching cells in tumor. Table S7 shows clinical parameters for patient samples. Table S8 shows up-regulated genes for each Seurat cluster in human integrated blood and initial tumor samples. Table S9 shows transcript AUC performance, delineated by melanoma patient sample. Table S10 shows significance measures calculated with COMET for all transcripts in sorting TM from non-TM CD8+ T cells in the blood of melanoma patients. Table S11 shows similarities and differences in COMET-identified markers to identify TM cells in mice with MC38 tumors compared with human melanoma patients. Table S12 lists the 16 transcripts that were significant in at least four patient samples, ordered by average ranking of AUC. Table S13 lists empirical significance values and 95% confidence intervals for the AUC, sensitivity, and specificity of featured gates in each patient sample. Table S14 lists sensitivity and specificity values for all possible transcriptional marker combinations of CCR7^low^, GYPC^low^, FLT3LG^low^, and LTB^low^, delineated by patient sample. Table S15 lists empirical significance values and 95% confidence intervals for the AUC, sensitivity, and specificity of featured gates in each patient sample using the intersection of GLIPH2 and iSMART.

## Supplementary Material

Table S1 shows up-regulated genes for each Seurat cluster in mouse integrated blood and MC38 tumor samples.

Table S2 shows up-regulated genes for TM and non-matching CD8^+^ T cells in the peripheral blood of mice with MC38 tumors.

Table S3 lists pathways and signatures enriched in TM and in non-matching CD8^+^ T cells from the peripheral blood of mice with MC38 tumors.

Table S4 shows significance measures calculated with COMET in sorting TM from non-matching CD8^+^ T cells in the blood of mice with MC38 tumors.

Table S5 shows the sensitivity and specificity of all possible gates made from combinations of NKG2D, CD39, and CX3CR1, measured by CITE-seq in mice.

Table S6 lists DE genes between TM cells in blood and blood-matching cells in tumor.

Table S7 shows clinical parameters for patient samples.

Table S8 shows up-regulated genes for each Seurat cluster in human integrated blood and initial tumor samples.

Table S9 shows transcript AUC performance, delineated by melanoma patient sample.

Table S10 shows significance measures calculated with COMET for all transcripts in sorting TM from non-matching CD8^+^ T cells in the blood of melanoma patients.

Table S11 shows similarities and differences in COMET-identified markers to identify TM cells in mice with MC38 tumors compared with human melanoma patients.

Table S12 lists the 16 transcripts that were significant in at least four patient samples, ordered by average ranking of AUC.

Table S13 lists empirical significance values and 95% confidence intervals for the AUC, sensitivity, and specificity of featured gates in each patient sample.

Table S14 lists sensitivity and specificity values for all possible transcriptional marker combinations of *CCR7*^low^, *GYPC*^low^, *FLT3LG*^low^, and *LTB*^low^, delineated by patient sample.

Table S15 lists empirical significance values and 95% confidence intervals for the AUC, sensitivity, and specificity of featured gates in each patient sample using the intersection of GLIPH2 and iSMART.
